# Supplementary material for: Incorporating hydrology into climate suitability models changes projections of malaria transmission in Africa
Source: Nat Commun. 2020 Aug 28;11:4353. doi: 10.1038/s41467-020-18239-5 (PMC7455692; doi:10.1038/s41467-020-18239-5)
Supplement: Supplementary file 1 — Supplementary Information [file 41467_2020_18239_MOESM1_ESM.pdf]

Supplementary Information

Incorporating hydrology into climate suitability models changes projections of malaria transmission in Africa

Smith et al.

Supplementary Notes (1-2)

Supplementary Tables (1-11)

Supplementary Figures (1-21)

Supplementary References

Supplementary Note 1.

Supplementary Note 1. Sensitivity analysis of model parameters

LIS-MAL estimates of hydro-climatic suitability for malaria transmission incorporate a minimum daily runoff threshold of 30 $m^3 s^{-1}$ (equivalent to ~1 mm depth across the grid cell per day) as a threshold for suitability, indicating sufficient water availability for a given grid cell. Unlike the use of a rainfall threshold, which implicitly incorporates multiple hydrological processes (e.g. infiltration, evaporation) within such a threshold, the runoff threshold represents a depth of surface water needed to form a suitable *Anopheles* mosquito breeding site. Thus, a lower threshold such as the daily 1 mm value used here, is more appropriate. The use of a threshold here reflects the relative large 0.5° grid size which aggregates multiple surface flows, from localised runoff to large river flows. Thus, such a threshold will be less important as hydro-climatic model spatial resolution improves in future. Prior to this, here we present a sensitivity analysis to evaluate the sensitivity of model hydro-climatic suitability estimates to this threshold.

The results from a single forcing model (r2: GFDL-ESM2M) were used for the sensitivity analysis, selected from the seven presented in the study as it yielded predictions within the extremes of the ensemble. Estimates for the historical period (1971-2005) were selected. Varying the 30 $m^3 s^{-1}$ threshold by 50% either way was considered to be an appropriate range of values; however, values beyond this range were also explored.

The runoff threshold was systematically adjusted between 1 $m^3 s^{-1}$ and 60 $m^3 s^{-1}$ at 1 $m^3 s^{-1}$ increments, yielding 60 model runs. The sensitivity of estimates of hydro-climatic suitability for malaria transmission to this threshold is presented in Supplementary Figure 14. Overall LIS-MAL suitability estimates were not sensitive to the runoff threshold within the 50% range: decreasing the runoff threshold by 50% increased the suitable area by 10%, while raising the threshold by 50% decreased the suitable area by just 6%. The suitability estimates were more sensitive at lower runoff thresholds (<10 $m^3 s^{-1}$) and more sensitivity was

observed in the 3-month categories of season length. The 1-3 month and 9-12 month categories were most sensitive within the 50% range.

Maps of hydro-climatic suitability estimates for selected runoff thresholds covering the range explored within the sensitivity analysis are presented in Supplementary Figure 15. The northern extent of transmission suitability does not change much within a wide range of thresholds; however, most noticeable is the change in year-round malaria suitability. At lower runoff thresholds, the LIS-MAL model identifies a much larger area of year-round suitability, extending to the fringes of transmission. The extent is more sensitive to runoff threshold in southern and eastern Africa; however, within a 50% range (represented by the 20, 30 and 40 $m^3 s^{-1}$ thresholds in Supplementary Figure 15) there is only a small degree of variability. Within central Africa, lower thresholds predict more widespread year-round malaria suitability, which may be more appropriate for these regions (see Supplementary Note 2).

For comparison, a similar sensitivity analysis was conducted for the rainfall threshold approach to climatic modelling of malaria transmission suitability. The 60 mm monthly rainfall threshold was taken as the base value in this case. The rainfall threshold was initially adjusted at 5 mm increments between 5 mm and 120 mm. Although this range matches the percentage variability investigated for the runoff threshold, it did not incorporate the range of rainfall thresholds reported in the literature (Supplementary Table 1). Thus, the upper range was extended to match the upper threshold in Supplementary Table 1.

While Supplementary Figure 16 indicates greater sensitivity at the extreme rainfall threshold values and for the monthly categories, the overall pattern is similar to that in Supplementary Figure 14. Decreasing the rainfall threshold by 50% increased the suitable area by 9%, while raising the threshold by 50% decreased the suitable area by 8%. Estimates of year-round malaria are sensitive to the rainfall threshold, even within the 50% range. Considering the full range of rainfall thresholds reported in the literature (reflecting the multiple and spatially variable hydrological processes aggregated into this threshold), there is considerable sensitivity to the rainfall threshold. It should be noted that the choice of the 60 mm rainfall threshold as a

base value here is somewhat arbitrary and conservative; selecting a higher value from Supplementary Table 1 would yield more sensitive suitability estimates. For a comparison of the effect of threshold variability with Supplementary Figure 15, see Figure 1 in the main text.

Supplementary Note 2.

Supplementary Note 2. Model validation against observations

Estimations of hydro-climatic suitability for malaria transmission are challenging to validate quantitatively for a number of reasons. First, the goal is to identify an envelope within which malaria transmission may occur; yet, numerous interventions have been undertaken to reduce the distribution of transmission over the last century[1]. For this reason, we focus our validation efforts on the pre-intervention map of Lysenko and Semashko[2] (~1900, reproduced in Supplementary Figure 17a), but recognise that substantial hydro-climatic changes will have taken place in the last 120 years and the exact locations of the boundaries in the pre-intervention map are likely subject to considerable uncertainty. Second, hydro-climatic suitability for malaria transmission does not necessarily mean that transmission will occur in those areas. There are numerous non-climatic controls on malaria transmission[3] and the absence of observed malaria transmission at a particular location does not necessarily mean that the model is incorrect. The anopheles inventory for sub-Saharan Africa (1898-2016) of Kyalo et al. (2017)[4] provides another validation map independent of climate (Supplementary Figure 17b) but is by its nature a discontinuous, positive point distribution. While continuous distribution maps of malaria transmission or vectors have been estimated (e.g. Bhatt et al. (2015)[5] and Wiebe et al. (2017)[6] (Supplementary Figure 17c-d), these rely in some part on gridded climate data for interpolation and cannot therefore serve for validation analysis independent of climate.

The pre-intervention map of Lysenko and Semashko[2] and anopheles presence map for sub-Saharan Africa (1898-2016) of Kyalo et al. (2017)[4] were each converted into a binary layer of presence/absence at the grid cell length of the hydro-climatic suitability model estimates. The mean of the seven hydro-climatic estimates for the historical period (1971-2005) was also converted into a binary layer using a minimum estimate of 0.5 months transmission as a threshold. Five validation metrics were calculated from the

resulting confusion matrix: (i) model accuracy (i.e. the proportion of all prediction that are correct); (ii) precision (i.e. the proportion of positive identifications that are correct); (iii) recall (i.e. the proportion of actual positives that are identified correctly); (iv) F1 score (the harmonic mean of precision and recall); (v) the false positive rate (i.e. the proportion of all actual negatives that are incorrectly identified as positive).

Results of the validation are provided in Supplementary Tables 10 and 11. All models performed similarly in terms of quantitative validation performance, especially given the uncertainty in validation sets. At the continental scale considered here this is not surprising given the strong link climatic controls on malaria transmission – there are many ways to model this relationship. In terms of both model accuracy and recall, the lower rainfall thresholds (Kiszewski[7], Ermert[8], Martens[9], Tanser[10] and Craig[11]) are best-performing, owing to the large areas estimated to be suitable in these models. By the same token, these same models report lower precision values and higher false positive rates. The converse is true regarding the higher rainfall thresholds (Garnham[12], Parham & Michael[13]) and the rainfall to potential evapotranspiration ratio of Lindsay[14]. The LIS-MAL and LIS-MAL with irrigation layers sit between these two extreme populations, scoring intermediate values for all metrics.

It is also important to examine the performance of models at the edges of transmission suitability, as these are precisely the areas that are most sensitive to changes in transmission potential due to climate change and where rivers may extend hydro-climatic suitability into the semi-arid regions that surround the core tropical malaria transmission area. We compared LIS-MAL and LIS-MAL + IRRA with an indicative rainfall-thermal model calculated from the methods of Tanser[10] qualitatively against published observations and descriptions of malaria and vector distribution, for four regions: The Sahel, Congo, Ethiopia and the Horn of Africa, and Southern Africa (Supplementary Figures 18 – 21).

Sahel Region: Sénégal

The LIS-MAL model highlights the northern border of Sénégal as suitable for malaria transmission year-round (further expanded with the inclusion of irrigation layers), while the Tanser rainfall-thermal model indicates a more linear north-south contrast, with the north being unsuitable year-round owing to low

rainfall (Supplementary Figure 9 shows that the area is well within suitable temperature ranges), the south being suitable for 3-6 months of the year and the middle just 1-3 months. Sénégal has a strong latitudinal pattern of malaria incidence in the country[15] and generally low and seasonal transmission, which follows the rainfall-threshold pattern. However, malaria transmission is also observed on the northern border beyond this threshold, driven by water availability in the Sénégal River basin with some vectors observed year-round[16]. Dia et al.[17] also noted year-round Anopheles larval breeding on the northern border with parasitic prevalence in schoolchildren indicative of a hypo to mesoendemic situation in the low lying river valley. Clearly, the spatial distribution of malaria transmission is more complex than national scale maps aggregated into health districts would suggest, thereby highlighting the difficulty of using such data sets to inform validation without further investigation. For instance, Diouf et al.[18] also note malaria cases in the northeast of the country; however, this is typically seasonal, except in particularly wet years. Meanwhile, Trape et al.[19] observe year-round malaria transmission in areas close to the Nema River owing to the year-round persistence of anopheles habitats (just north of The Gambia); however, owing to the scale of the feature, this was not resolved in the current version of LIS-MAL. Niang et al.[20] note that the year-round presence of anopheles larvae in streams does not result in year-round malaria transmission in central Sénégal. The complexity of the spatial pattern of malaria transmission is better represented by LIS-MAL in Sénégal, especially the hydro-climatic suitability of areas in the north of the country; however, the year-round transmission may be an over-estimate. The Tanser model more correctly identifies the limited seasonal transmission that extends across the southern two-thirds of the country.

Sahel Region: Mali

Each model broadly agrees with observations in Mali, where malaria suitability is focused in the south-west of the country. However, unlike rainfall-based estimations, LIS-MAL indicates that the Niger River forms a suitable transmission corridor that extends much further north. Observed Demographic and Health Survey data[21] indicate this northward extension to some extent, though survey sparsity makes firm conclusions problematic. More recently, Coulibaly et al.[22] observed malaria transmission over 7 months of the year where the Tanser rainfall-based model would indicate transmission 4-6 months and LIS-MAL indicated 6-9

month transmission. Coulibaly et al.[22] specifically reported the effect of the Yame River in supporting transmission during periods of low discharge when rainfall was reduced.

Sahel Region: Nigeria

Models are generally in agreement with the finding that the whole of Nigeria is malarious[23], though it seems that a focus in the centre-north of the country is not seen in hydro-climatic suitability estimates. While Tolulope[24] reported variable patterns in malaria morbidity each year, this northern focus was consistent. A further focus in the west of the country is captured in each hydro-climatic model.

Sahel Region: Niger

Again, models agree on the suitability for malaria transmission at the southern border with Nigeria, which matches observations. Labbo et al.[25] note the near year-round presence of vectors at two villages close to the Niger River which is indicated by LIS-MAL but not rainfall threshold models. The more seasonal transmission reported[26] at the central southern border was also recreated well by both approaches.

Sahel Region: The Nile

LIS-MAL predicts much more extensive hydro-climatic suitability for malaria transmission in Sudan than rainfall-based models. Malik and Khalafalla[27] note the presence of malaria throughout Sudan, including hypo-endemic areas on the far north attributed to association with rivers. Ageep et al.[28] confirm the year-round presence of anopheles larvae close to the Nile. In Gezira state year-round malaria cases were observed[29] (2007-2016), with an increase between July and October. Further north, malaria has been well-known in Egypt since ancient times[30], with proven vectors present as far north as the Nile delta[31]. Shousha[32] reported year-round occurrence of invading *An. gambiae* in 1944 prior to a successful eradication campaign in 1945. Thus, while there are currently no local cases of malaria, it is certainly hydro-climatically suitable for malaria and areas close to water bodies are thought to be of high risk of future malaria outbreaks[33].

Democratic Republic of the Congo (DRC)

All hydro-climatic representations of malaria transmission indicate that the Democratic Republic of the Congo (DRC) is situated within the most widespread focus of suitability (Supplementary Figure 19). While quantitative validation suggest that all models perform equally well here, it is nonetheless worthwhile to examine the differences in the model estimates.

A comparison of LIS-MAL and Tanser model estimates reveals three key differences:

(1) The LIS-MAL estimates produce more complex spatial patterns;
(2) The LIS-MAL estimates indicate that a much higher area of the country is suitable for malaria transmission year-round, with river corridors serving as foci for this sustained transmission suitability;
(3) The LIS-MAL model estimates that substantial areas are only hydro-climatically suitable for <3 months of the year as the threshold discharge is not met in these areas

Unfortunately, there is a high level of uncertainty regarding the actual spatial distribution of malaria in the DRC, with the fewest data points per land area of any country[34]. The inverse distance weighted spatially interpolated model of Messina et al.[34] based on Demographic Health Survey data highlights clusters of transmission that was observed to be more associated with social factors (e.g. wealth, community use of bed nets). Janko et al.[35] also note a positive association between agriculture and malaria risk in the DRC. Nevertheless, the generally higher prevalence of malaria parasitaemia in the north was not observed in either model; indeed, the LIS-MAL model suggests <3 months suitable for transmission in some of these areas. Malaria transmission in the DRC is more widespread than suggested by the LIS-MAL model as seen in recent studies[36]. This suggests that the areas where LIS-MAL suggests <3 month transmission suitability in the DRC and surrounding countries are an under-estimate, most likely associated with the relatively coarse scale of hydrological modelling.

Ethiopia

Using information presented in NMCT et al.[37], all models correctly identified the highlands as malaria-free and longer transmission seasons in west. However, large areas of the country are arid with malaria near water, features not captured in rainfall threshold modelling but identified in LIS-MAL models, at least where water bodies were detectable at the spatial resolution of the model (Supplementary Figure 20). For example, LIS-MAL mapped the observed long transmission seasons associated with rivers in the southeast in the Wabi Shebel and Genale Dawa catchments and LIS-MAL+IRRA additionally mapped areas suitable for annual transmission associated with irrigation development, for example along the Awash River and in the Rift Valley. LIS-MAL also highlights Lake Abbe in the Afar Depression (Ethiopia-Djibouti border) as suitable for transmission. The saline nature of this environments highlights a limitation of the hydrological modelling employed herein; however, Kyalo et al.[4] indicates that anophelines are observed at this location.

Horn of Africa

Noor et al.[38] assembled observations of parasite prevalence and showed the south of Somalia as a focus of transmission, particularly around the Juba and Shabelle rivers identified by LIS-MAL, However, both LIS-MAL and Tanser models failed to identify the low transmission areas in the north. Geostatistical modelling of parasite prevalence[38] suggested environmental factors unmeasured at the 1 km resolution of their analysis may be a factor, such as artificial breeding sites in bore holes, wells, dams and seasonal streams and the same is would to apply to LIS-MAL and Tanser models. In addition, neither LIS-MAL nor rainfall threshold models account for potential adaptations of vectors in the region to extreme heat and aridity (e.g. Omer and Cloudsley-Thompson[39]) that may support low-levels of transmission.

South Africa

All models agree that the focus of malaria transmission suitability in South Africa is at the border with Mozambique, as confirmed by field observations[40,41] (Supplementary Figure 21). LIS-MAL generally indicates reduced overall transmission suitability in eastern South Africa, when compared with the Tanser rainfall approach. However, the extent of suitability in LIS-MAL stretches much further west, along river corridors. While there are no contemporary observations of malaria in the Orange Free State (see also the

Lysenko and Semashko[2] pre-intervention (~1900) map of malaria endemicity in Africa), a survey of malaria and mosquitos in 1921 reported that areas of malaria were observed "*present only to a very slight degree, and are mainly, if not entirely, confined to the immediate vicinity of rivers, such as the Vaal, Vet, Riet and Caledon*"[42]. The malaria suitability of the corridor along the Orange River was suggested to be flood driven[42]. The much expanded area of transmission suitability estimated when mapped irrigation is added (Supplementary Figure 21c) may be an overestimate of suitability, since the same report[42] notes that malaria was practically unknown in Cape Province; however, to the extent of irrigation during the pre-intervention period remains unknown.

Supplementary Tables

Supplementary Table 1. Summary of rainfall thresholds used in indices of climatic suitability of malaria transmission.

Rainfall Criterion	Equivalent rainfall per month	Reference
>80 mm per month maintained for 5 months (or 3 months at high temperatures)	80 mm	Craig *et al.* (1999)[11]<br>Lindsay and Martens (1998)[43]<br>Martens *et al.* (1999)[44]
>1.5 mm per day	46.5 mm	Martens *et al.* (1995)[9]
Ratio of rainfall to potential evapotranspiration >0.5 over 5 months	-	Martens *et al.* (1997)[45]<br>Lindsay *et al.* (1998)[14]
>60 mm per month for 3 months plus single catalyst month of >80 mm	60 mm (plus catalyst month of 80 mm)	Tanser *et al.* (2003)[10]
10 mm per month for temporary water bodies (suggest permanent bodies independent of rainfall)	10 mm	Kiszewski *et al.* (2004)[7]
5 mm per day	152 mm	Garnham (1948)[12]<br>Hay *et al.* (2002)[46]
5 mm per day with upper threshold of 50 mm per day	155 mm <Rain <1520 mm	Parham and Michael (2010)[13]
10 mm of rain per dekad with upper threshold of 500 mm per dekad. Fuzzy suitability actually decreases to 0 mm.	31 mm<Rain<1550 mm	Ermert *et al.* (2011)[8]

Supplementary Table 2. Climate projections downscaled with EC-EARTH3-HR and corresponding year of exceeding 1.5, 2 and 4°C warming. From Alfieri *et al.*[47].

	Forcing Model	Ensemble Member	1.5°C	2°C	4°C
1	IPSL-CM5A-LR	r1i1p1	2015	2030	2068
2	GFDL-ESM2M	r1i1p1	2040	2055	2113
3	HadGEM2-ES	r1i1p1	2027	2039	2074
4	EC-EARTH	r12i1p1	2019	2035	2083
5	GISS-E2-H	r1i1p1	2022	2038	2102
6	IPSL-CM5A-MR	r1i1p1	2020	2034	2069
7	HadCM3LC	r1i1p1	2003	2020	2065

Supplementary Table 3. Total areas estimated to be hydro-climatically suitable for malaria transmission for each hydrological representation and thermal response model for each time period. Areas suitable for > 3 months (i.e. stable areas) are also calculated.

	Total Area (Mn km^2)					Stable Area (Mn km^2)			
	1971-2005	2011-2040	2041-2070	2071-2100		1971-2005	2011-2040	2041-2070	2071-2100
Hydrological representation									
LIS-MAL	15.66	15.56	15.74	15.82		11.73	11.56	11.73	11.80
LIS-MAL + Irrigation	17.43	Not used for future time periods				14.12	Not used for future time periods		
Tanser[10]	19.04	19.02	19.17	19.20		14.84	14.76	14.89	14.87
Kiszewski[7]	24.26	24.14	24.25	23.23		19.84	19.98	19.96	19.47
Ermert[8]	20.87	20.90	21.05	20.88		17.21	17.33	17.52	17.52
Martens[9]	19.85	19.85	19.94	19.91		15.90	15.97	16.13	16.17
Craig[11]	17.89	17.84	18.05	18.16		13.07	12.71	12.78	12.84
Garnham[12]	13.51	13.38	13.62	13.96		4.00	3.76	3.79	4.33
Parham & Michael[13]	13.33	13.19	13.43	13.79		3.70	3.51	3.53	4.02
Lindsay[14]	15.21	15.55	15.92	16.01		10.06	10.22	10.51	10.37
Thermal response model									
Mordecai[48]	17.89	17.84	18.05	18.16		13.07	12.71	12.78	12.84
Mara[11]	17.48	17.61	17.92	18.14		12.28	12.30	12.57	12.78
Parham & Michael[13]	16.26	17.00	17.62	18.00		10.21	11.08	11.96	12.50

Supplementary Table 4. Summary of model agreement for different hydrological representations across all 7 forcing models. Model agreement stated as the area with majority model agreement (i.e. ≥4 models) as a percentage of the total area that is classified as suitable (i.e. by ≥1 model).

	Percentage area with majority agreement							
	Stable (>3 months suitable)				Unstable (1-3 months suitable)			
	1971-2005	2011-2040	2041-2070	2071-2100	1971-2005	2011-2040	2041-2070	2071-2100
Hydrological representation								
LIS-MAL	76.64	72.27	70.79	69.30	12.96	14.03	13.51	12.03
LIS-MAL + Irrigation	81.29	Not used for future time periods			11.95	Not used for future time periods		
Tanser[10]	88.35	87.05	87.92	82.77	12.15	16.63	12.12	10.14
Kiszewski[7]	90.60	88.72	88.74	85.61	10.28	11.45	8.23	3.50
Ermert[8]	90.13	89.10	91.02	86.69	11.33	13.75	11.19	8.20
Martens[9]	89.05	88.50	90.27	85.34	13.47	14.99	11.63	9.14
Craig[11]	63.80	82.72	82.03	75.43	10.93	10.52	9.03	8.32
Garnham[12]	45.43	43.95	44.53	39.46	13.87	14.18	15.87	13.60
Parham & Michael[13]	44.44	43.17	44.09	38.77	15.30	15.15	17.35	14.28
Lindsay[14]	81.39	79.29	79.00	74.03	10.85	10.85	9.33	9.90
Thermal response model								
Mordecai[48]	63.80	82.72	82.03	75.43	10.93	10.52	9.03	8.32
Mara[11]	81.53	81.56	81.61	74.71	8.89	9.79	9.14	8.65
Parham & Michael[13]	73.15	75.74	80.06	74.29	5.62	7.07	7.93	8.85

Supplementary Table 5. Changes in total and stable areas (suitable for > 3 months) between time periods.

	Change in Total Area (Mn km^2)				Change in Stable Area (Mn km^2)		
	2011-2040	2041-2070	2071-2100		2011-2040	2041-2070	2071-2100
Hydrological representation							
LIS-MAL	-0.10	0.08	0.16		-0.17	0.00	0.08
Tanser[10]	-0.02	0.12	0.16		-0.07	0.05	0.03
Kiszewski[7]	-0.13	-0.01	-1.03		0.14	0.12	-0.37
Ermert[8]	0.02	0.18	0.00		0.12	0.31	0.31
Martens[9]	0.01	0.10	0.06		0.07	0.22	0.26
Craig[11]	-0.05	0.16	0.27		-0.36	-0.29	-0.22
Garnham[12]	-0.14	0.10	0.45		-0.24	-0.21	0.32
Parham & Michael[13]	-0.14	0.10	0.46		-0.18	-0.17	0.33
Lindsay[14]	0.34	0.71	0.80		0.15	0.44	0.30
Thermal response model							
Mordecai[48]	-0.05	0.16	0.27		-0.36	-0.29	-0.22
Mara[11]	0.14	0.45	0.66		0.02	0.29	0.49
Parham & Michael[13]	0.74	1.35	1.74		0.87	1.75	2.29

Supplementary Table 6. Areas experiencing an increase and decrease in malaria suitability in future projections. Note that the net value includes areas with an average change of <1 month and only where the signal to noise ratio >0.5 and thus does not match the value in Supplementary Table 5.

	2011-2040			2041-2070			2071-2100		
	Increasing	Decreasing	Net	Increasing	Decreasing	Net	Increasing	Decreasing	Net
Hydrological representation (Mn km^2)									
LIS-MAL	0.52	1.06	-0.54	0.88	1.26	-0.38	1.28	1.43	-0.15
Tanser[10]	0.45	0.48	-0.04	0.85	2.06	-1.21	1.78	4.16	-2.38
Kiszewski[7]	1.29	0.74	0.55	1.92	2.19	-0.27	1.82	4.55	-2.73
Ermert[8]	0.70	0.54	0.16	1.06	2.26	-1.20	1.63	3.68	-2.05
Martens[9]	0.51	0.50	0.01	0.84	2.02	-1.18	1.44	3.91	-2.46
Craig[11]	0.37	0.65	-0.28	0.63	2.39	-1.76	1.76	3.74	-1.98
Garnham[12]	0.15	0.71	-0.56	0.33	1.32	-0.99	1.24	1.82	-0.58
Parham & Michael[13]	0.16	0.61	-0.45	0.37	1.29	-0.92	1.24	1.66	-0.42
Lindsay[14]	1.01	0.43	0.58	1.87	0.51	1.36	2.38	1.61	0.77
Thermal response model (Mn km^2)									
Mordecai[48]	0.37	0.65	-0.28	0.63	2.39	-1.76	1.76	3.74	-1.98
Mara[11]	0.52	0.58	-0.06	0.78	1.86	-1.09	1.79	2.81	-1.02
Parham & Michael[13]	1.62	0.49	1.12	1.99	1.00	0.99	1.91	1.26	0.65

Supplementary Table 7. Increase and decrease in malaria suitability 'month-areas' (in millions mo km^2) in future projections where the areas used to calculate totals in Supplementary Table 6 are multiplied by the number of months change in malaria climatic suitability to provide a clearer indication of the magnitude of changes.

	2011-2040			2041-2070			2071-2100		
	Increasing	Decreasing	Net	Increasing	Decreasing	Net	Increasing	Decreasing	Net
Hydrological representation									
LIS-MAL	0.70	0.53	0.16	1.32	1.28	0.04	2.10	2.15	-0.05
Tanser[10]	0.67	0.24	0.43	1.21	1.03	0.18	1.31	2.08	-0.78
Kiszewski[7]	1.49	0.37	1.12	1.91	1.92	-0.01	2.25	6.83	-4.58
Ermert[8]	1.05	0.27	0.78	1.44	1.13	0.31	1.46	3.47	-2.01
Martens[9]	0.77	0.25	0.52	1.40	1.01	0.39	1.34	1.95	-0.61
Craig[11]	0.56	0.32	0.23	0.96	1.20	-0.24	1.41	1.87	-0.46
Garnham[12]	0.22	0.35	-0.13	0.46	0.66	-0.20	1.24	0.91	0.33
Parham & Michael[13]	0.19	0.31	-0.11	0.43	0.65	-0.21	1.43	0.83	0.60
Lindsay[14]	0.54	0.21	0.32	2.35	0.37	1.98	3.56	2.41	1.16
Thermal response model									
Mordecai[48]	0.56	0.32	0.23	0.96	1.20	-0.24	1.41	1.87	-0.46
Mara[11]	1.16	0.29	0.87	1.94	0.93	1.01	2.10	1.40	0.70
Parham & Michael[13]	4.04	0.25	3.80	6.26	0.50	5.76	6.83	0.63	6.20

Supplementary Table 8. Total populations (Mn) and children under 5 years old within areas of climatic suitability for malaria transmission in Africa (>1 month) and climatic suitability for stable malaria transmission (>3 months) from WorldPop 2015 data.

	> 1 month		>3 months	
	All	< 5 s	All	< 5 s
Hydrological representation				
LIS-MAL	822	135	619	100
Tanser[10]	962	158	752	124
Kiszewski[7]	1096	174	940	155
Ermert[8]	1033	166	864	143
Martens[9]	991	161	810	134
Craig[11]	919	151	655	108
Garnham[12]	704	117	217	34
Parham & Michael[13]	695	115	201	31
Lindsay[14]	774	131	506	85
Thermal response model				
Mordecai[48]	919	151	655	108
Mara[11]	867	144	594	98
Parham & Michael[13]	770	129	507	85

Supplementary Table 9. Total populations (Mn) and children under 5 years old within areas of climatic suitability for malaria transmission in Africa (>1 month) and climatic suitability for stable malaria transmission (>3 months). Population figures are calculated from the mid-point of each time period using the Medium Variant figures and the mean of each of the seven GCM forcings.

Population (Mn)	Number of months suitable	LIS-MAL				Tanser[10]			
		1971-2005	2011-2040	2041-2070	2071-2100	1971-2005	2011-2040	2041-2070	2071-2100
All	>1 month	822	1079	2062	3117	962	1264	2391	3554
<5s only	>1 month	135	159	227	257	158	188	265	295
All	>3 months	619	801	1561	2384	752	998	1962	2990
<5s only	>3 months	100	117	171	195	124	150	219	249

Supplementary Table 10. Comparison of hydro-climatic model accuracy and precision for the validation data sets.

	Accuracy		Precision	
	Pre-intervention (~1900) malaria[2]	Anopheles inventory (1898-2016)[4]	Pre-intervention (~1900) malaria[2]	Anopheles inventory (1898-2016)[4]
LIS-MAL	0.80	0.61	0.96	0.41
LIS-MAL + Irrigation	0.83	0.58	0.95	0.40
Tanser[10]	0.87	0.59	0.95	0.40
Kiszewski[7]	0.85	0.45	0.85	0.34
Ermert[8]	0.88	0.55	0.93	0.38
Martens[9]	0.88	0.57	0.95	0.40
Craig[11]	0.85	0.60	0.96	0.41
Garnham[12]	0.74	0.65	0.98	0.43
Parham & Michael[13]	0.74	0.65	0.98	0.43
Lindsay[14]	0.79	0.64	0.99	0.42

Supplementary Table 11. Comparison of hydro-climatic model recall, F1 statistic and false positive rate for the validation data sets.

	Recall		F1		False Positive Rate	
	Pre-intervention (~1900) malaria[2]	Anopheles inventory (1898-2016)[4]	Pre-intervention (~1900) malaria[2]	Anopheles inventory (1898-2016)[4]	Pre-intervention (~1900) malaria[2]	Anopheles inventory (1898-2016)[4]
LIS-MAL	0.76	0.83	0.85	0.55	0.09	0.48
LIS-MAL + Irrigation	0.81	0.87	0.87	0.55	0.11	0.53
Tanser[10]	0.87	0.95	0.91	0.57	0.12	0.55
Kiszewski[7]	0.96	1.00	0.90	0.50	0.48	0.77
Ermert[8]	0.91	0.98	0.92	0.55	0.19	0.62
Martens[9]	0.89	0.96	0.92	0.56	0.14	0.58
Craig[11]	0.84	0.92	0.89	0.57	0.09	0.52
Garnham[12]	0.67	0.75	0.79	0.55	0.03	0.39
Parham & Michael[13]	0.66	0.74	0.79	0.54	0.03	0.39
Lindsay[14]	0.72	0.80	0.83	0.56	0.03	0.43

Supplementary Figures

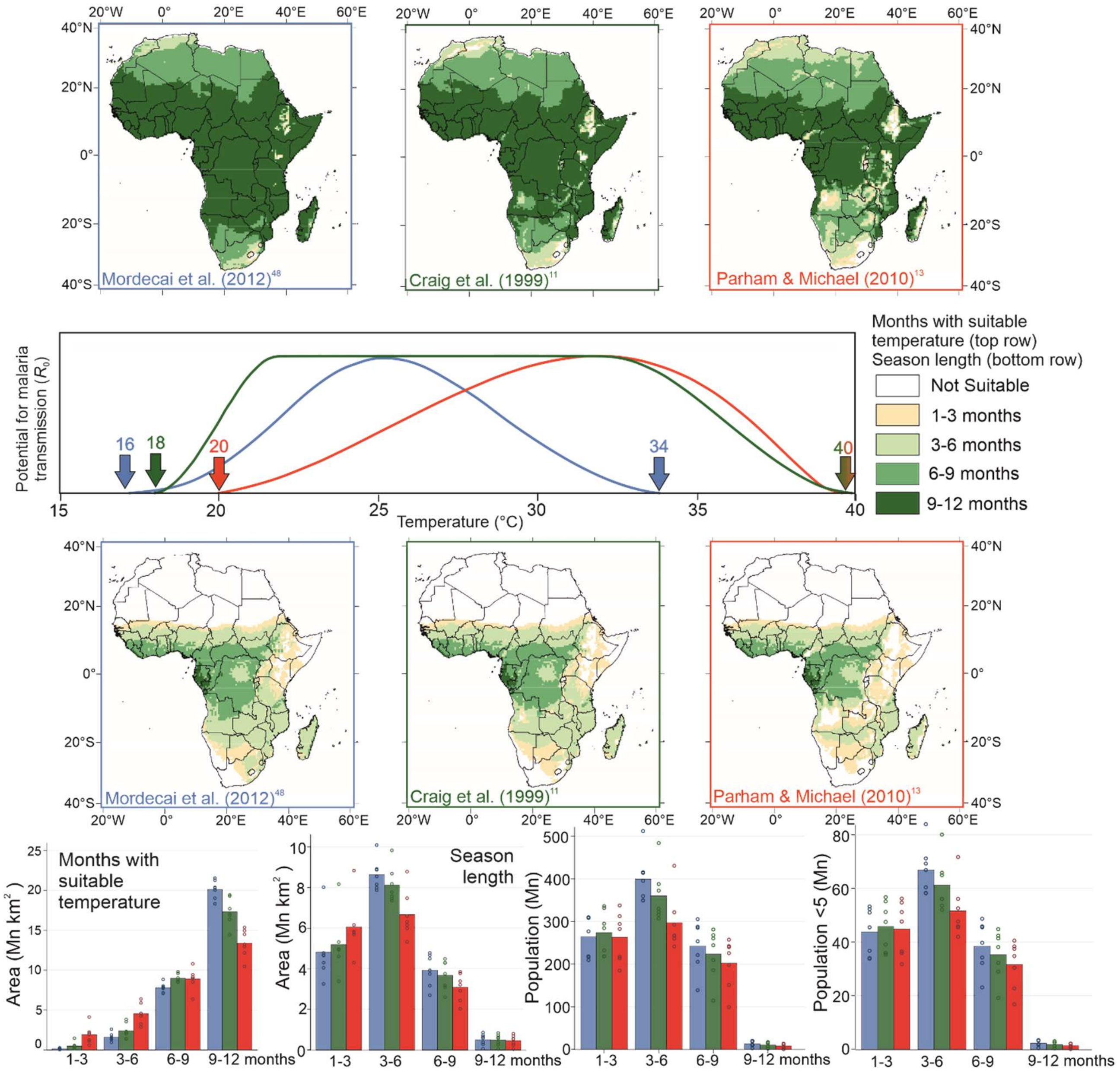

Supplementary Figure 1. The effect of thermal response curve on estimates of climatic suitability for malaria transmission in Africa. The individual thermal response curves are presented on the second row with the ranges indicated. Note that the model of Craig *et al.*[11] also includes a frost criterion excluding areas with a minimum mean monthly temperature of -4°C. The number of months per year within each temperature curve is presented on the top row. The climatic suitability is presented on the third row with an 80 mm rainfall threshold applied and continuous months counted to represent transmission season length. Areas within 3-month bands are calculated for both temperature suitability and season length on the bottom row alongside total population and number of under 5s in each 3-month band. Bars indicate mean of model estimates (*n*=7); points indicate individual model estimates.

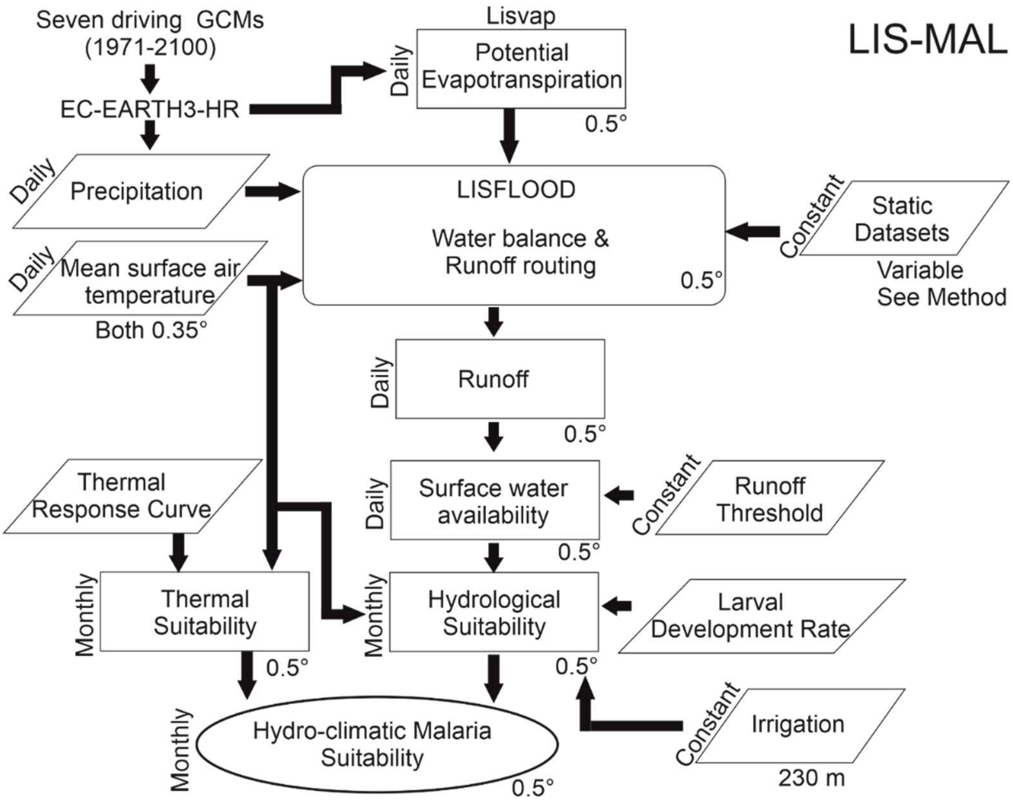

Supplementary Figure 2. LIS-MAL model structure. The spatial resolution of each input of calculation is given in the bottom right corner of each box, while the temporal variability is indicated along the left side.

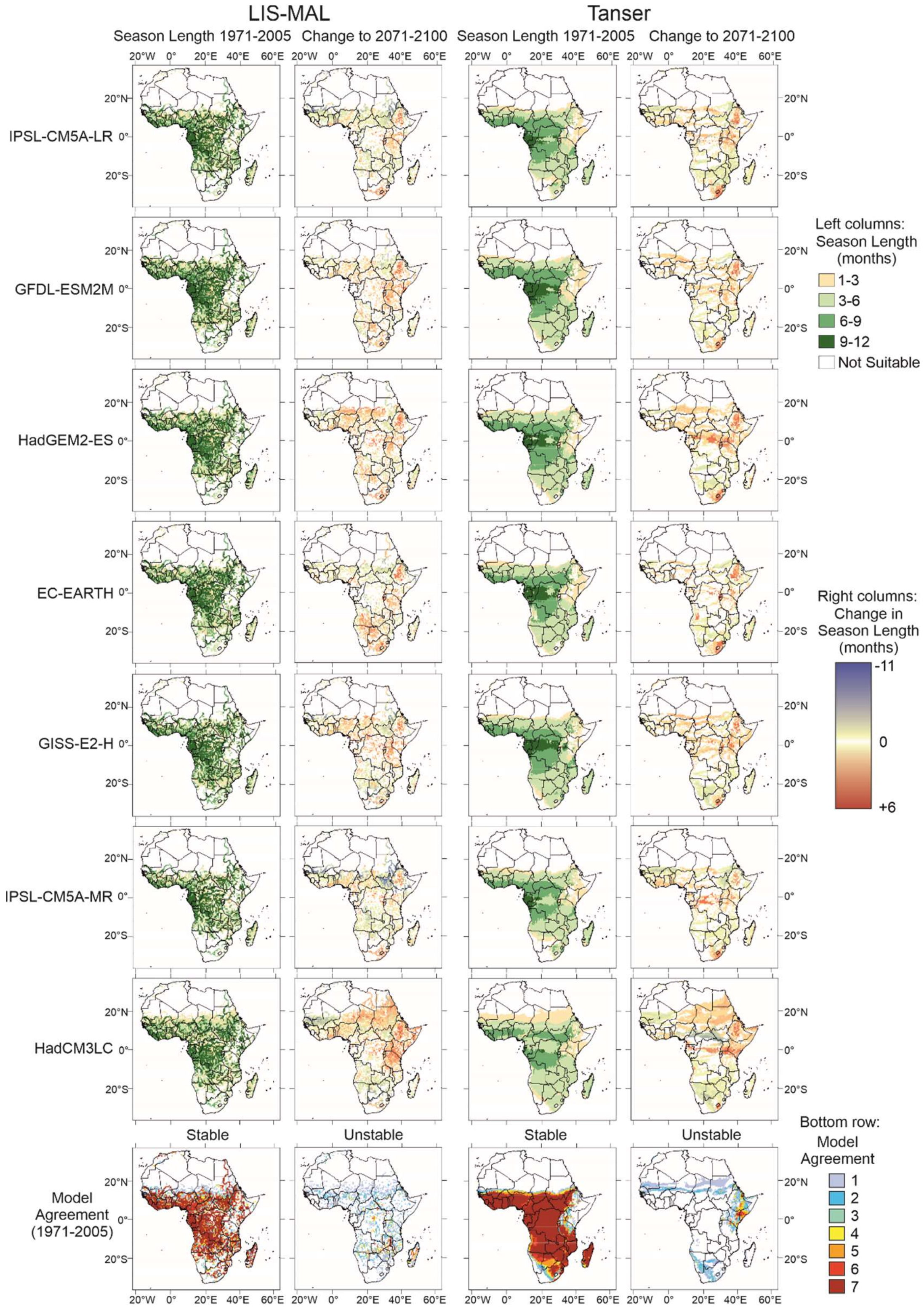

Supplementary Figure 3. Individual general circulation models. Comparison of estimates of climatic suitability for malaria transmission in Africa using the Tanser[10] rainfall threshold and LIS-MAL (thermal response in both is represented using the Mordecai *et al.*[48] curve for consistency). Hydro-climatic suitability for malaria transmission (1971-2005) estimated using climatic models from each of the seven downscaled general circulation models detailed in Supplementary Table 2 (left columns for both hydrological representations). Change in malaria hydro-climatic suitability predicted by Lisflood and Tanser models between 1971-2005 and 2071-2100 for each forcing model (right columns). Model agreements for both stable (> 3 months) and unstable malaria transmission are presented on the bottom row (see also Supplementary Table 5).

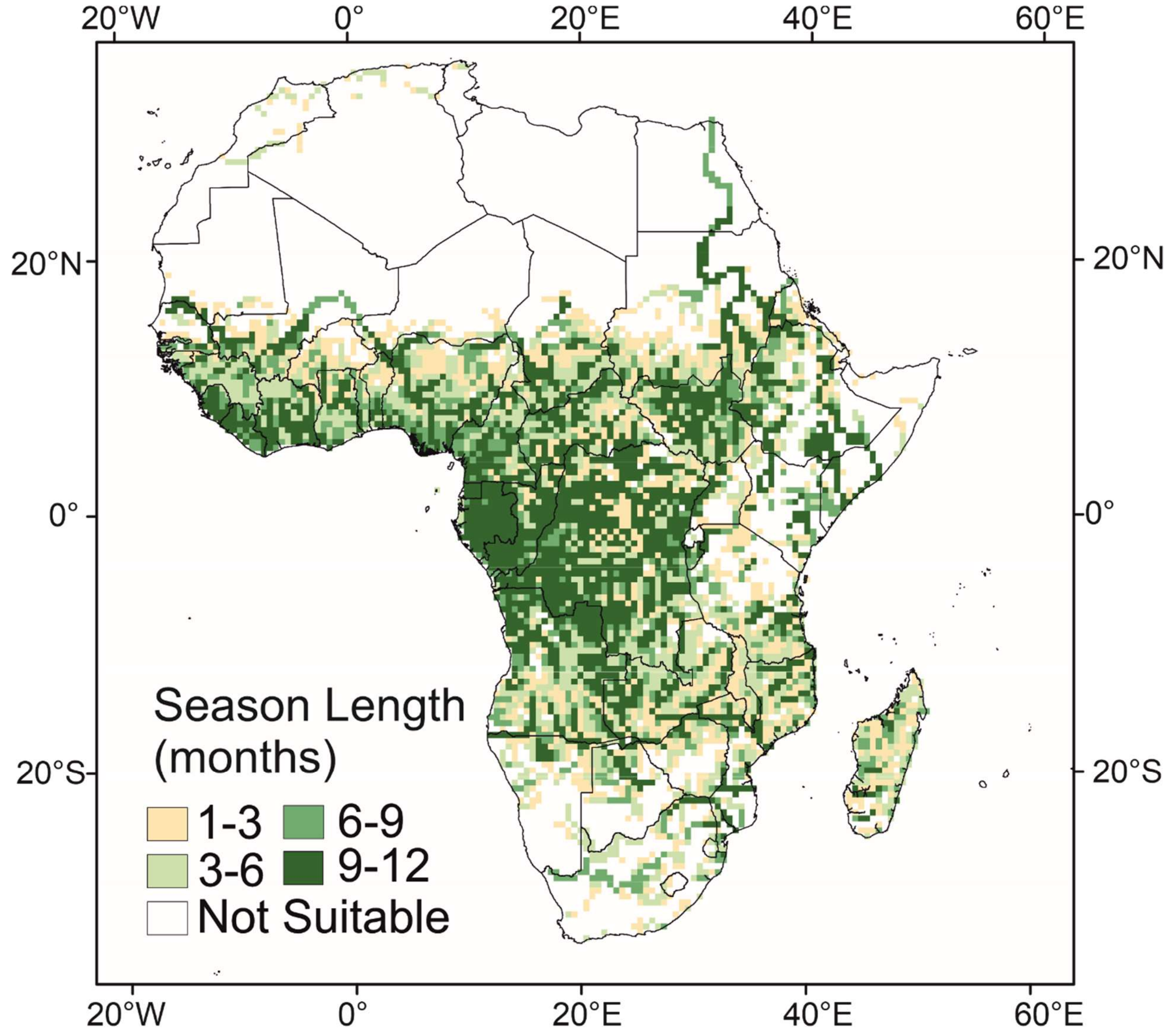

Supplementary Figure 4. Estimate of current climatic suitability for malaria transmission. Detailed reproduction of the estimate of climatic suitability for malaria transmission in Africa using LIS-MAL with the Mordecai *et al.*[48] temperature range.

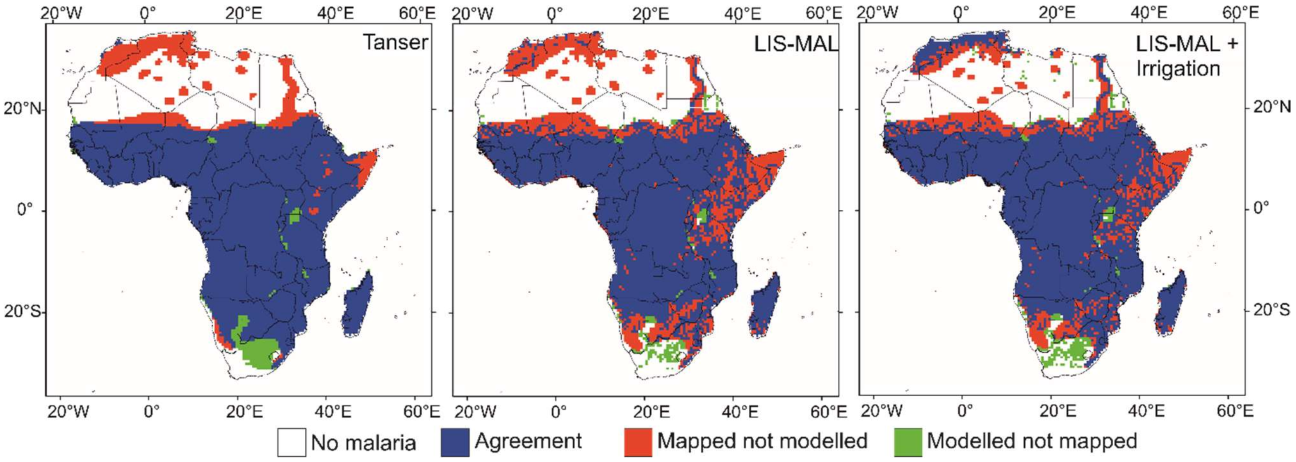

Supplementary Figure 5. **Validation of present day estimates.** Comparison of LIS-MAL and Tanser[10] (hydro-)climatic malaria suitability model estimates with the pre-intervention malaria map of Lysenko and Semashko[2]. More detailed validation is presented in Supplementary Note 2.

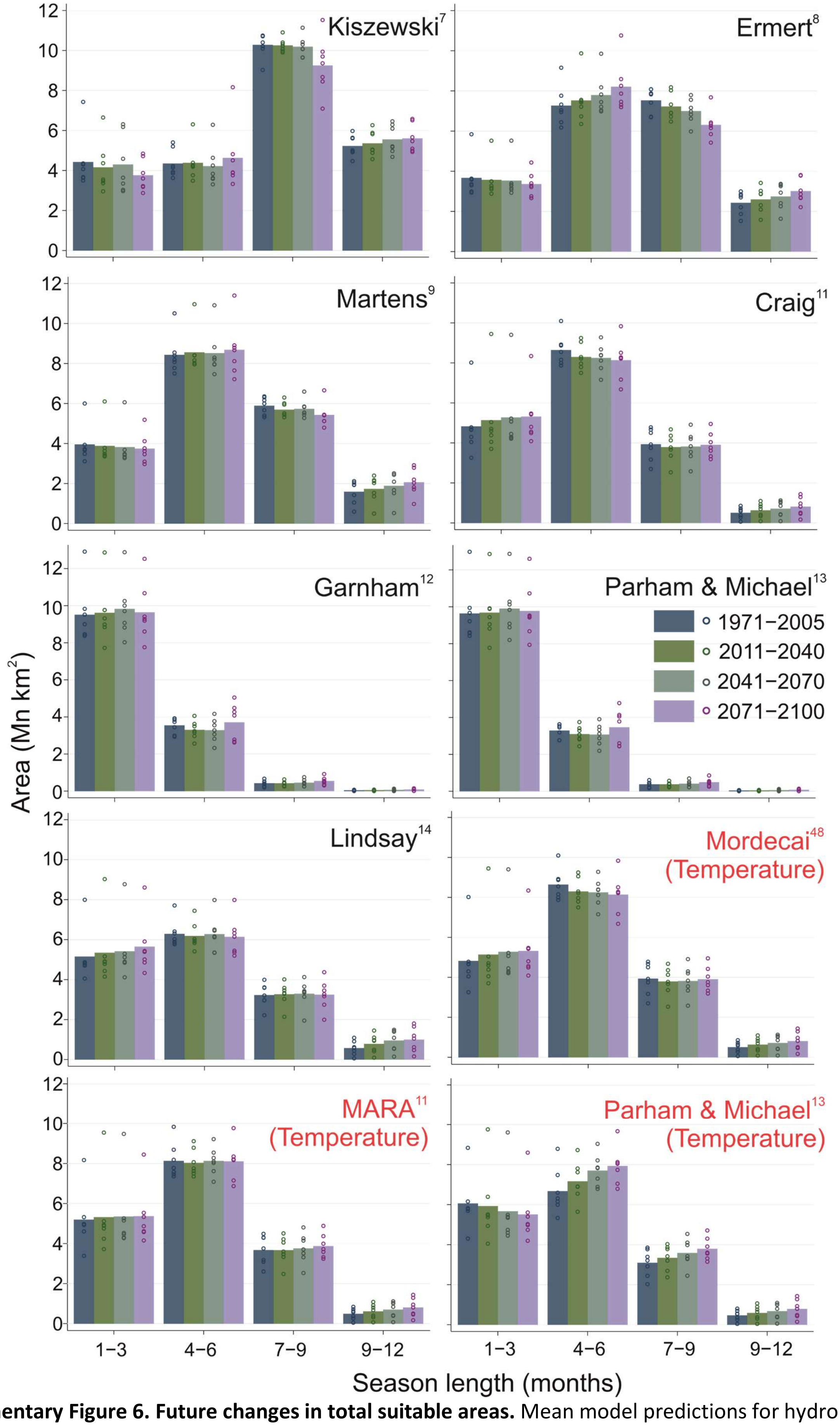

Supplementary Figure 6. Future changes in total suitable areas. Mean model predictions for hydrological representations not shown in Figure 3 at each time step with a summary of areas in 3-month categories. Bars indicate mean of model estimates (*n*=7); points indicate individual model estimates.

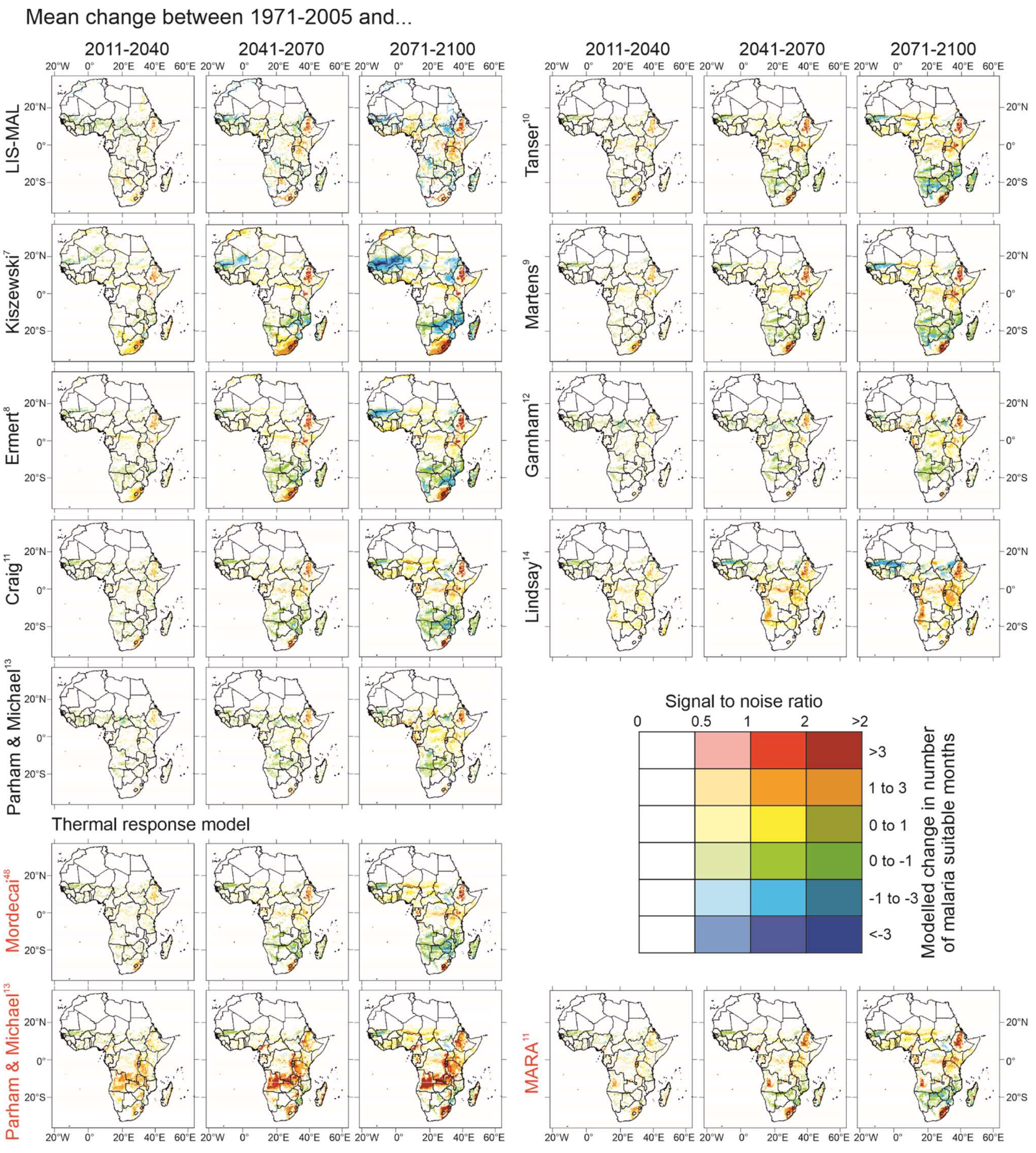

Supplementary Figure 7. Changes in duration of malaria transmission climatic suitability between each period for each hydrological and thermal representation. Different saturations indicate the signal-to-noise ratio across the seven projections, with the noise defined as the standard deviation of estimates across the projections, as per Figure 3.

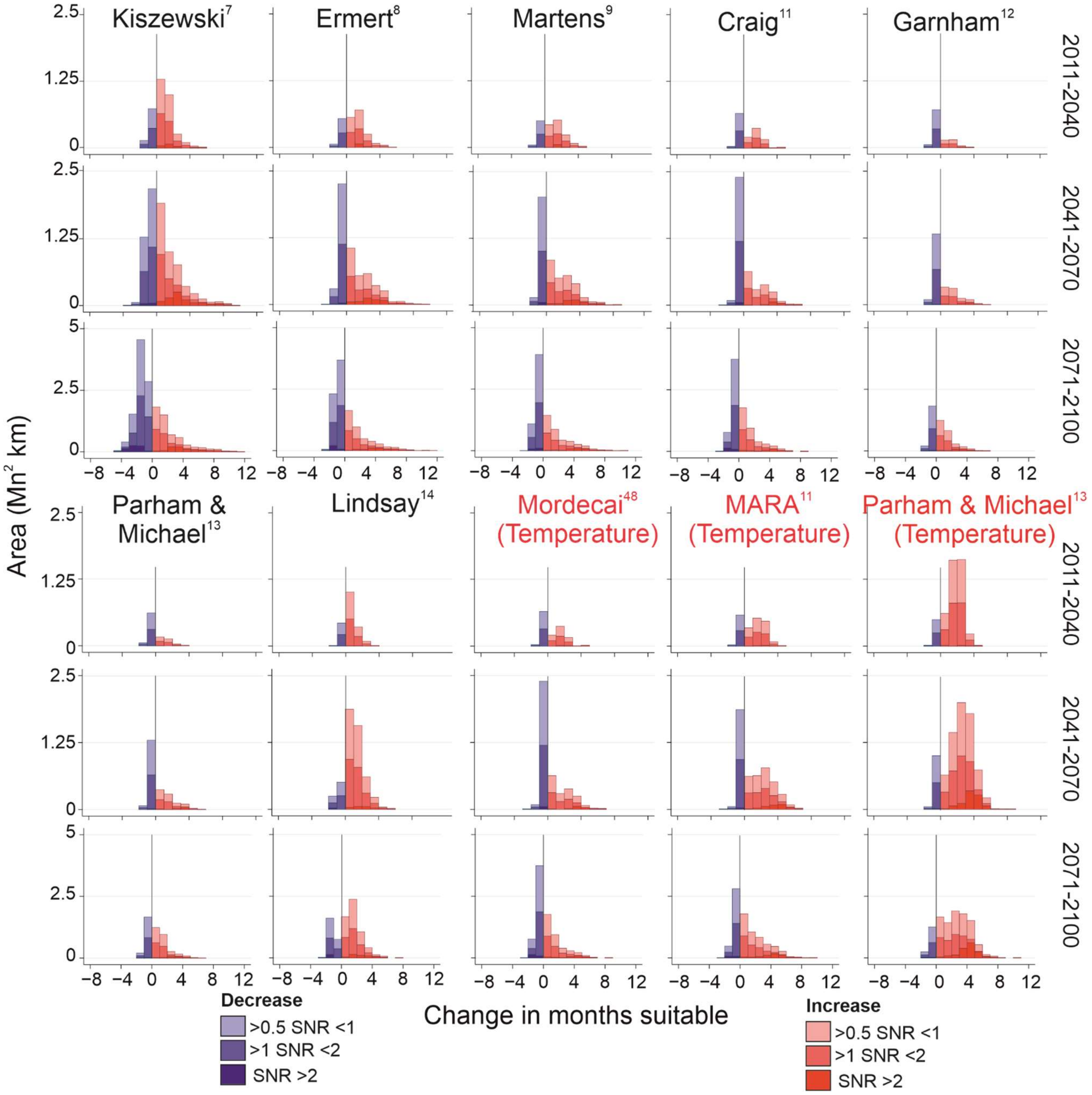

Supplementary Figure 8. Areas with changing malaria transmission suitability. Stacked histograms showing changes number of months climatically suitable for all periods and both hydrological and thermal representations split into categories of signal-to-noise ratio as per Figure 3.

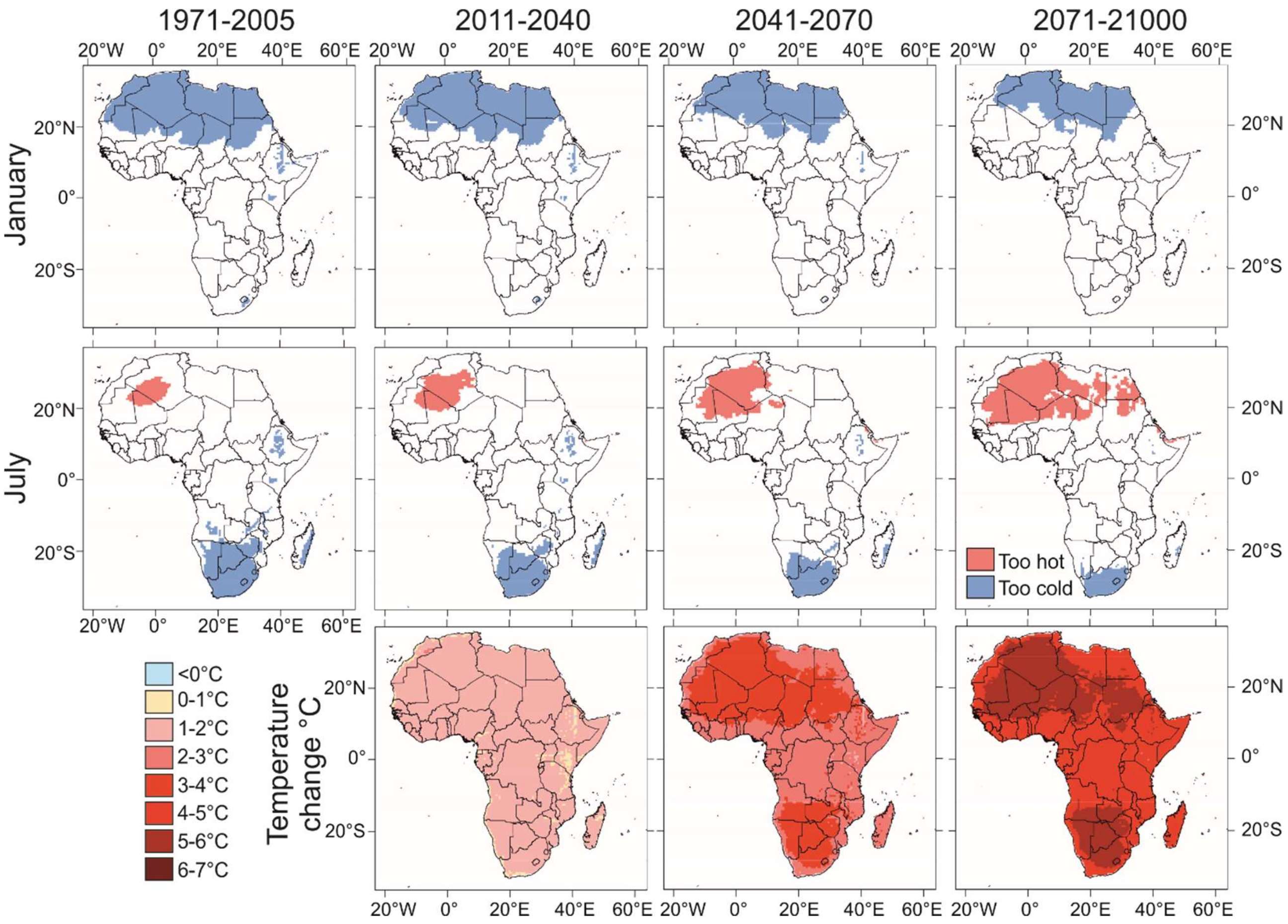

Supplementary Figure 9. Projected changes in temperature for each period. Areas too cold (blue) or too hot (red) on the Mordecai *et al.*[48] curve are shown for January and July. Average changes in monthly temperature between each period and 1971-2005 shown on the bottom row.

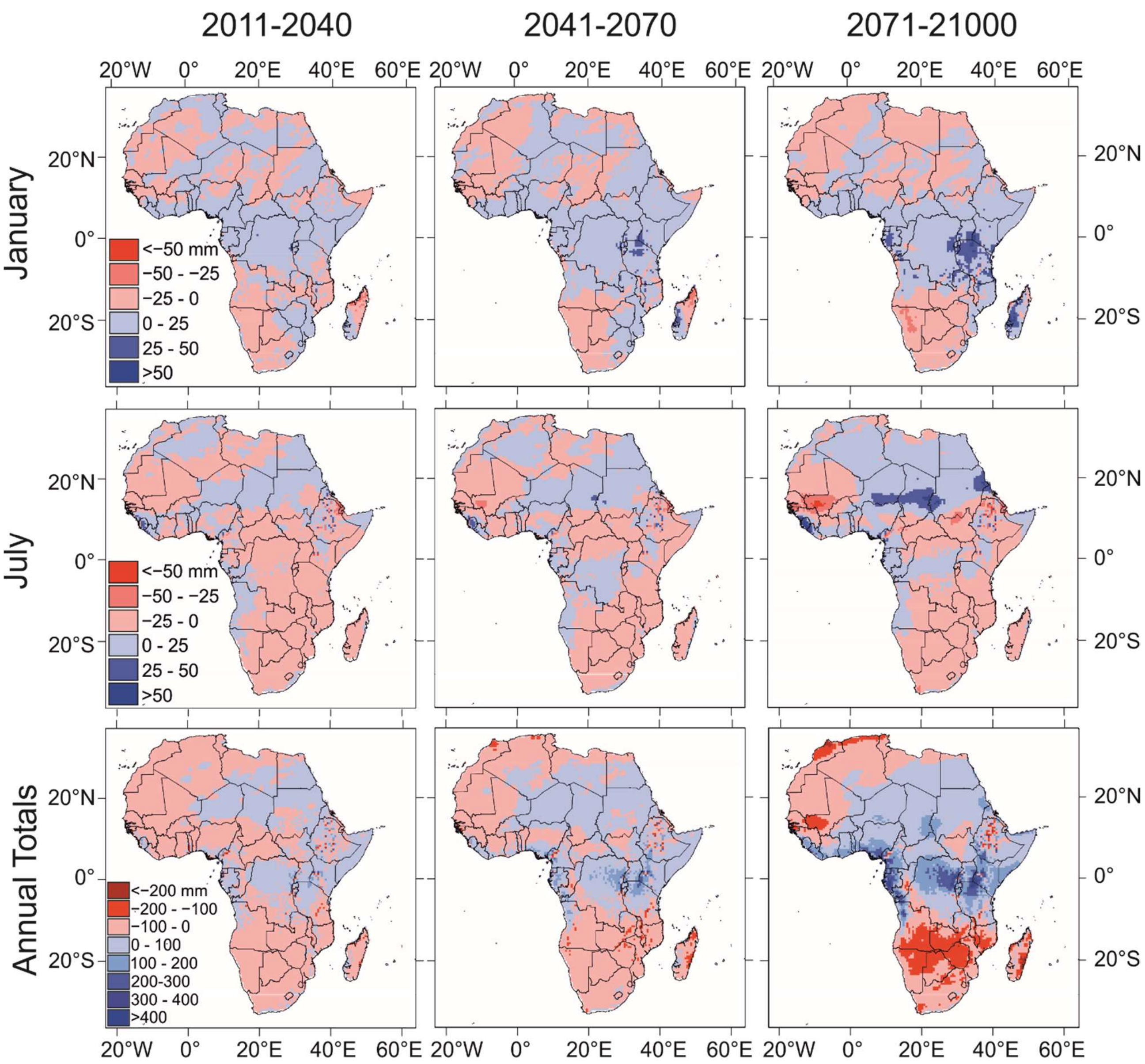

Supplementary Figure 10. Projected changes in rainfall for each period. Changes for January and July are shown. Changes in annual totals between each period and 1971-2005 are shown on the bottom row.

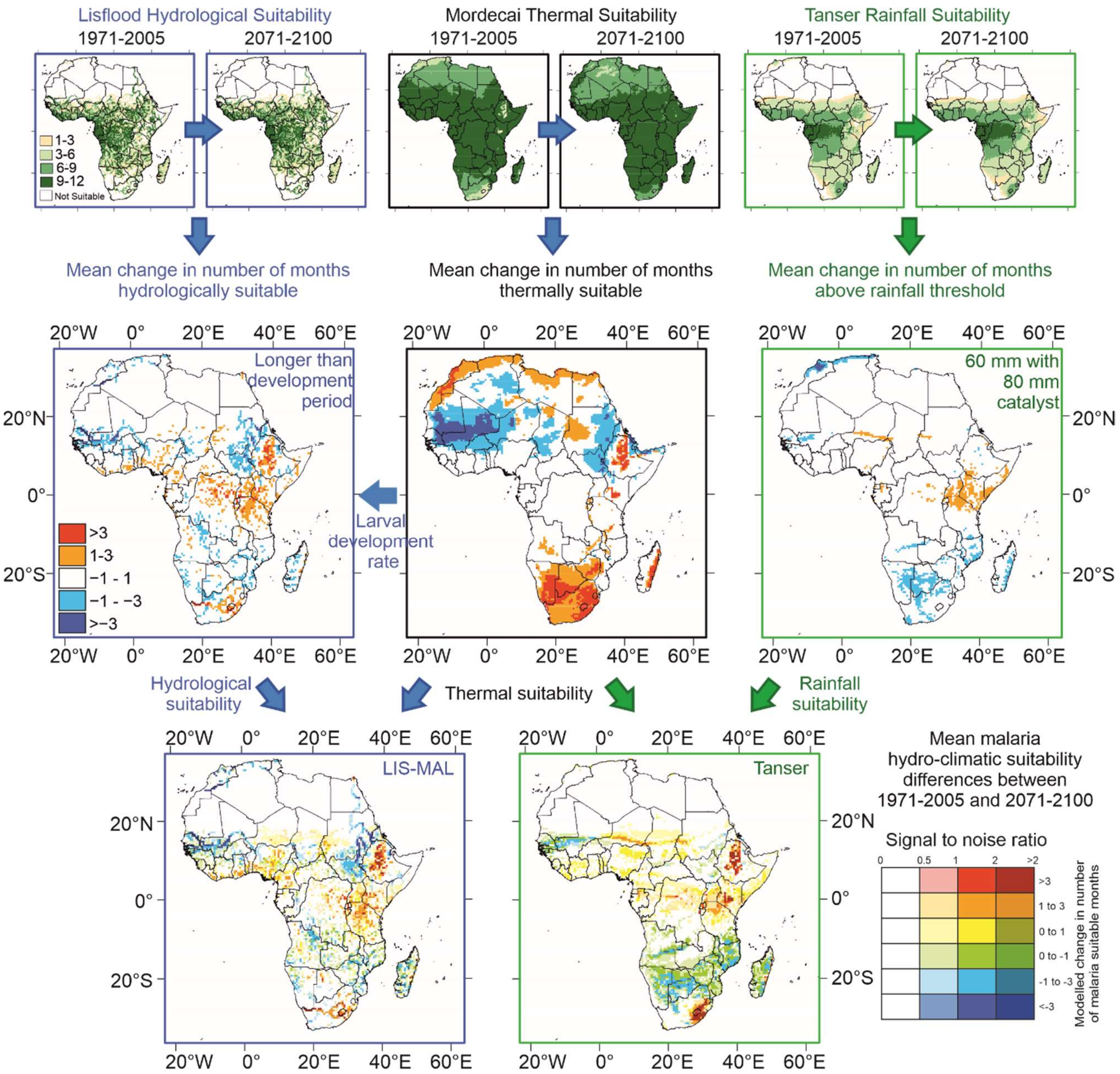

Supplementary Figure 11. Drivers of projected changes. Hydrological and thermal drivers of projected changes in malaria hydro-climatic suitability for both the LIS-MAL approach and Tanser rainfall threshold-based estimates.

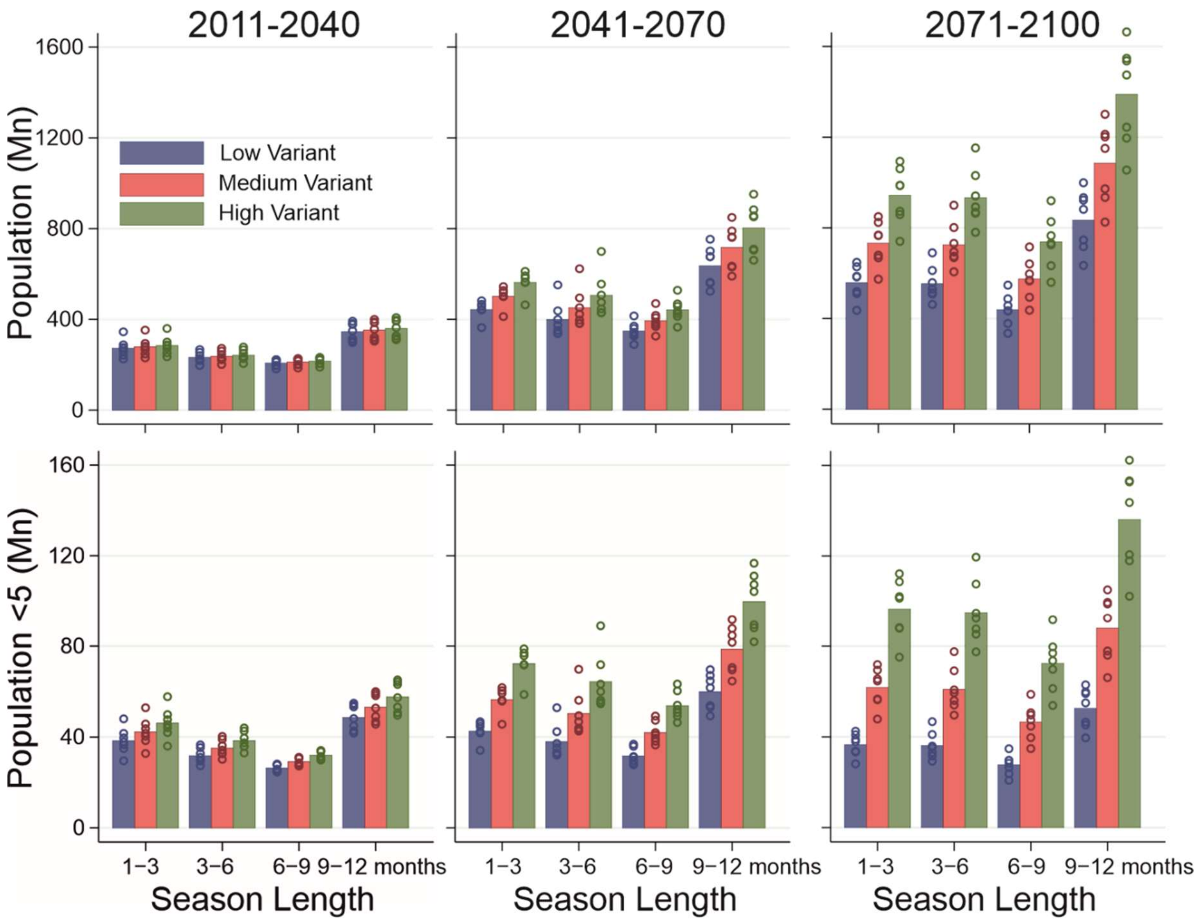

Supplementary Figure 12. Comparison of UN population variants. The effect of UN population variant (High, Medium or Low) on future population predictions of malaria suitability in Africa for the LIS-MAL hydro-climatic model. Results from each of the climate projections are displayed separately with the bar representing the mean value (*n*=7).

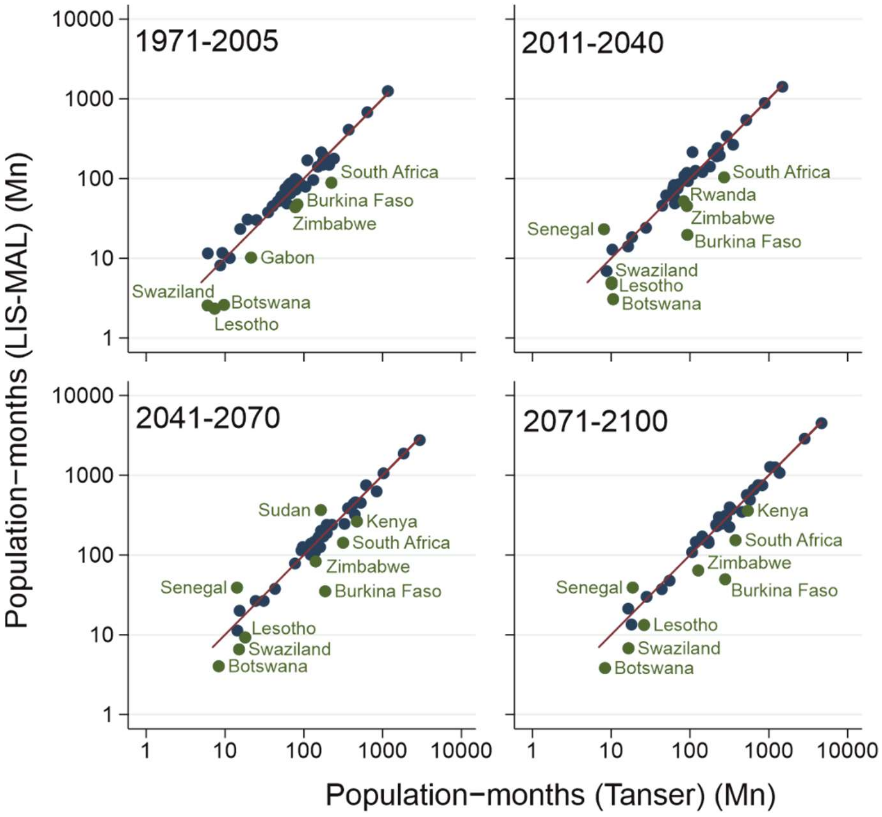

Supplementary Figure 13. Country-by-country comparison of population-months. Comparison is presented for each period according to estimates from both LIS-MAL and the Tanser rainfall threshold (i.e. population exposed multiplied by the number of additional months of exposure). The 1:1 line is shown in red. Individual countries are highlighted when a >50% difference in predictions is observed.

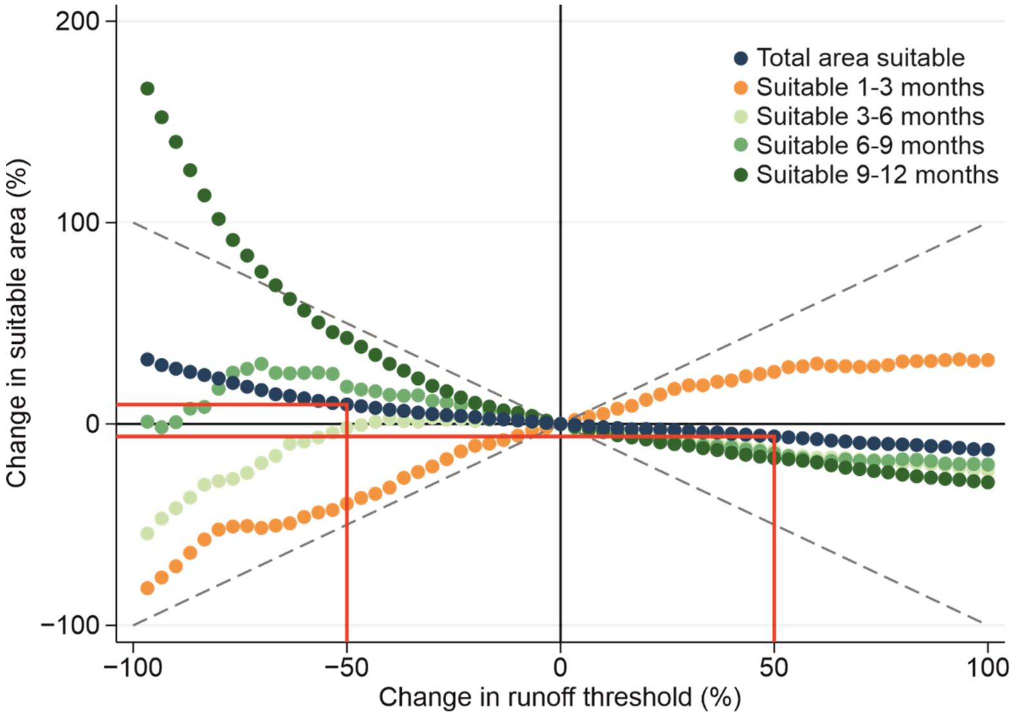

Supplementary Figure 14. Sensitivity analysis for the LIS-MAL runoff threshold.

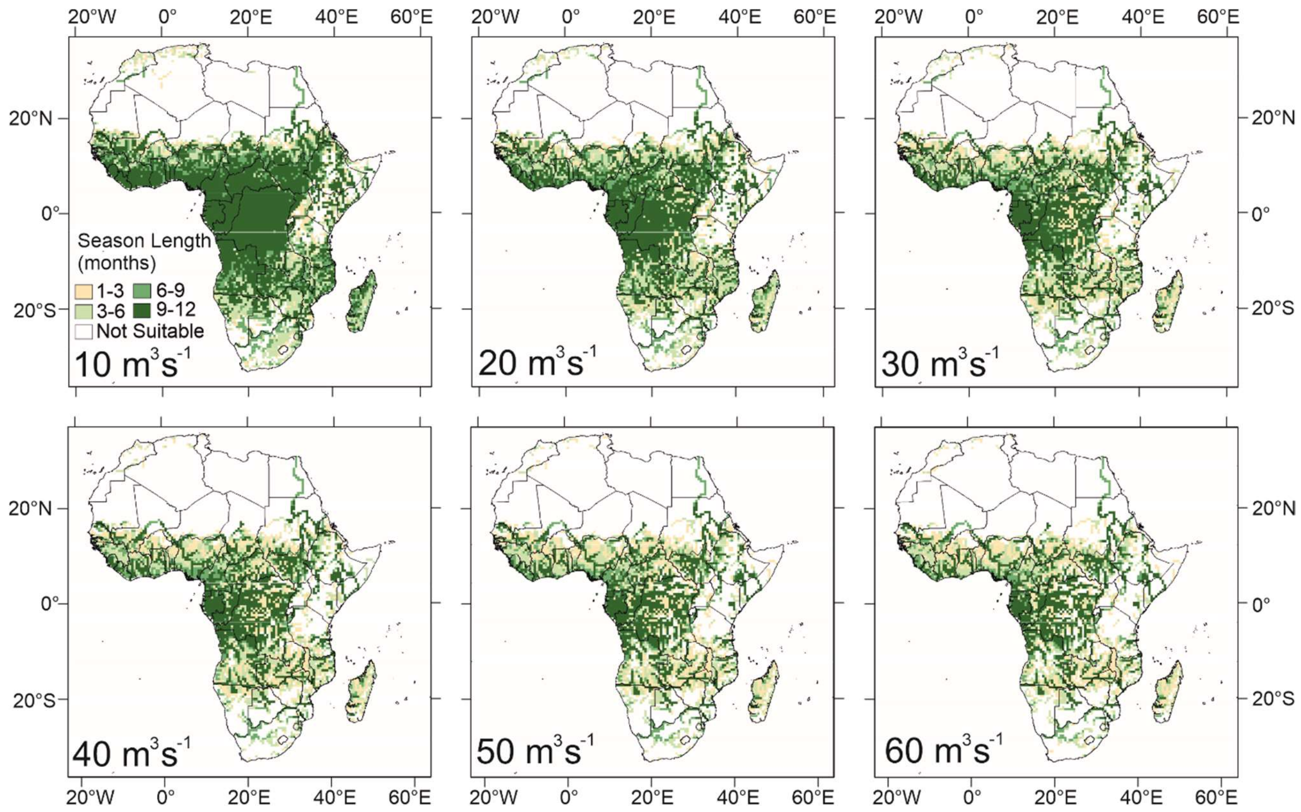

Supplementary Figure 15. Runoff threshold effect on estimates. Variability of LIS-MAL hydro-climatic malaria suitability estimates across the range of runoff thresholds used within the sensitivity analysis.

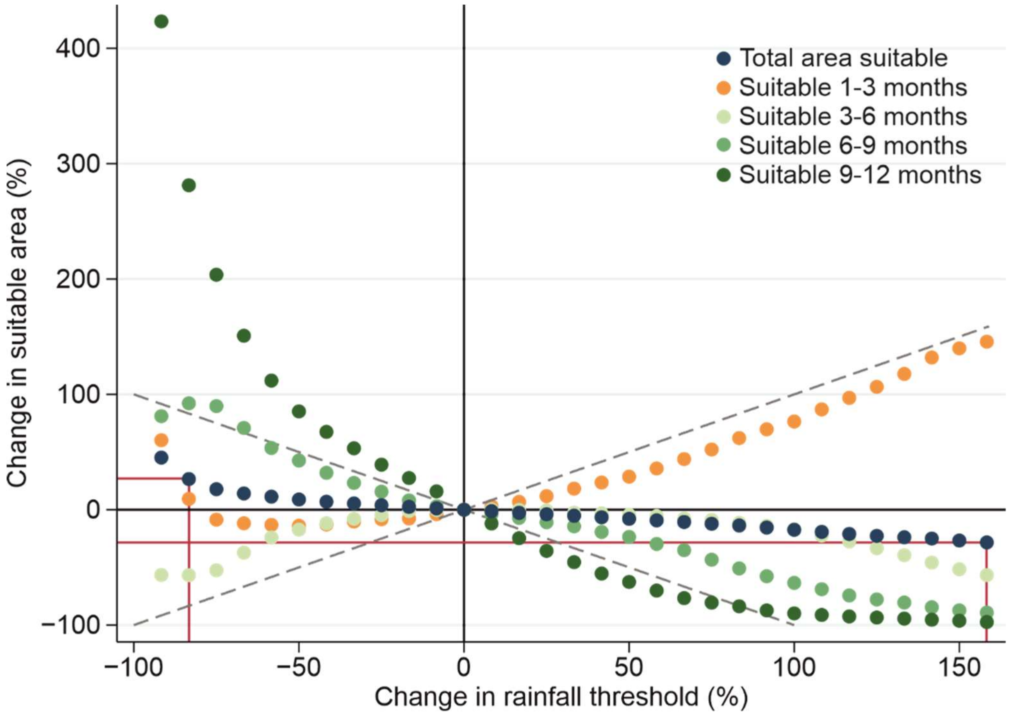

Supplementary Figure 16. Sensitivity analysis for rainfall threshold approaches of estimating climatic suitability for malaria transmission.

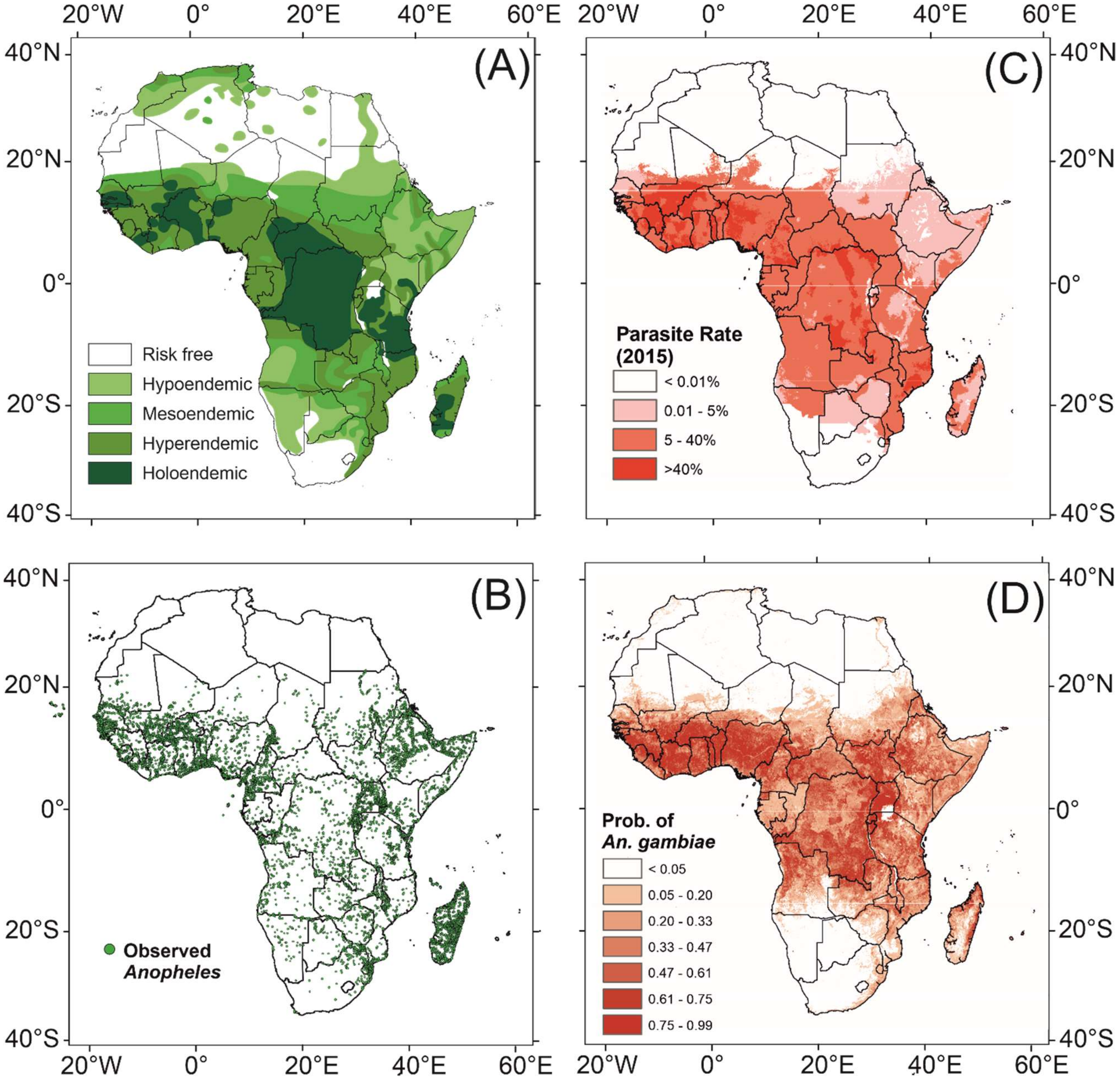

Supplementary Figure 17. Validation datasets available for comparison with the hydro-climatic estimate of environmental suitability for malaria transmission. Observed or mapped data are in the left column in green and are used in the validation presented herein. Modelled estimates are shown in red in the right column and are not compared quantitatively here owing to their dependence on climate data. Lysenko and Semashko (1968)[2] pre-intervention (~1900) map of malaria endemicity in Africa (A). Kyalo et al. (2017)[4] anopheles inventory of sub-Saharan Africa (1898-2016) displaying all observations of anophelines (B). Malaria Atlas Project modelled estimates of parasite rate (C) from Bhatt et al. (2015)[5] for 2015. Wiebe et al. (2017)[6] estimate of the mean modelled relative probability of occurrence of *Anopheles gambiae* (D).

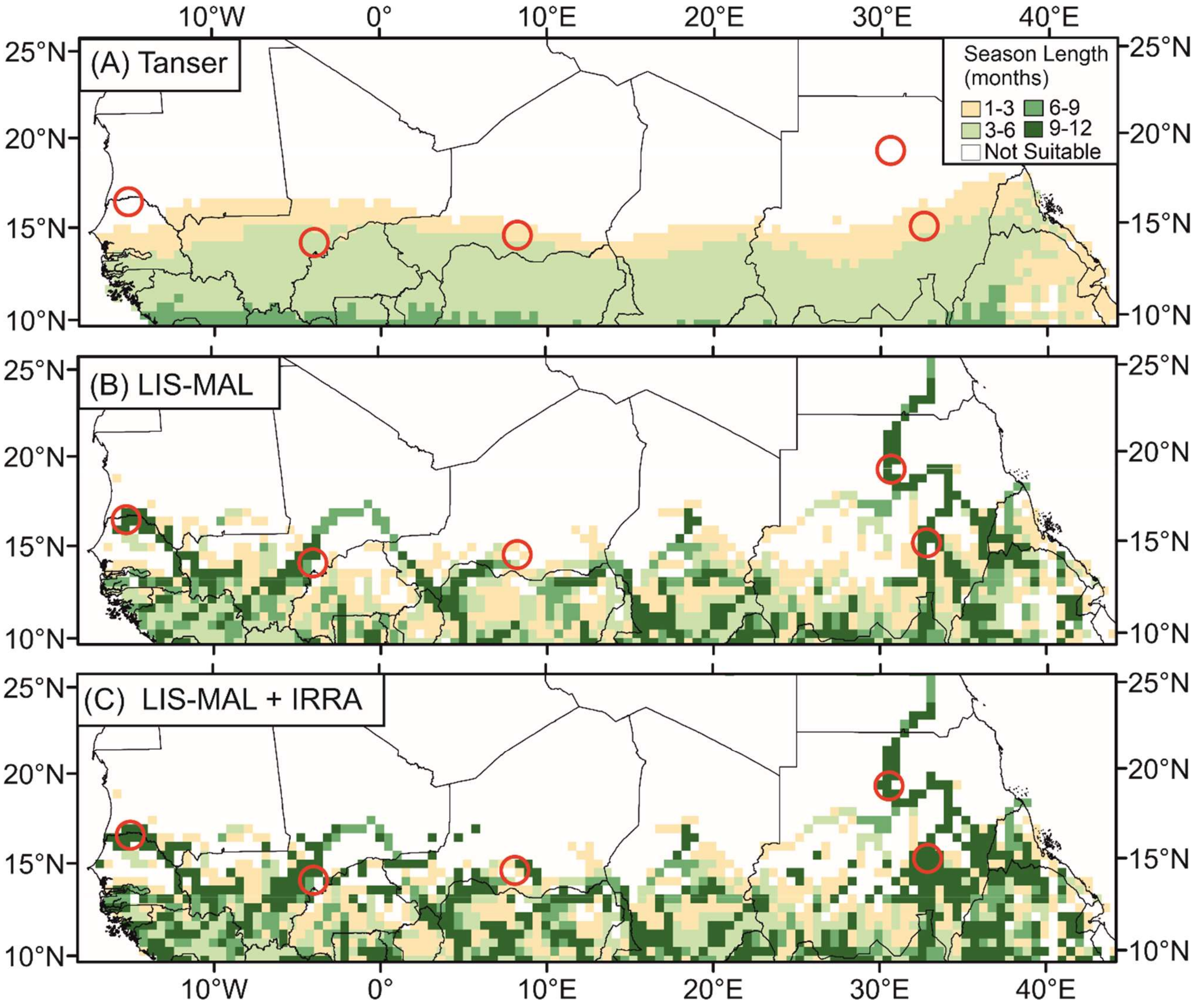

Supplementary Figure 18. **Comparison of hydro-climatic model estimates across the Sahel region.** Red circles indicate areas of discussion in Supplementary Note 2.

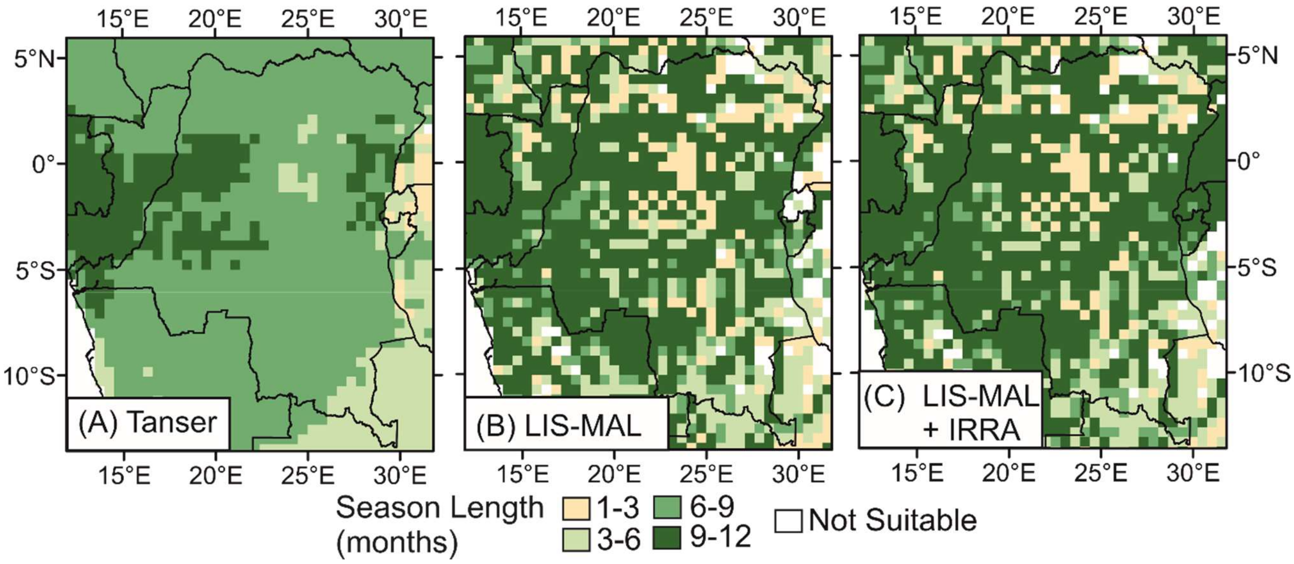

Supplementary Figure 19. **Comparison of hydro-climatic model estimates for the Democratic Republic of the Congo.**

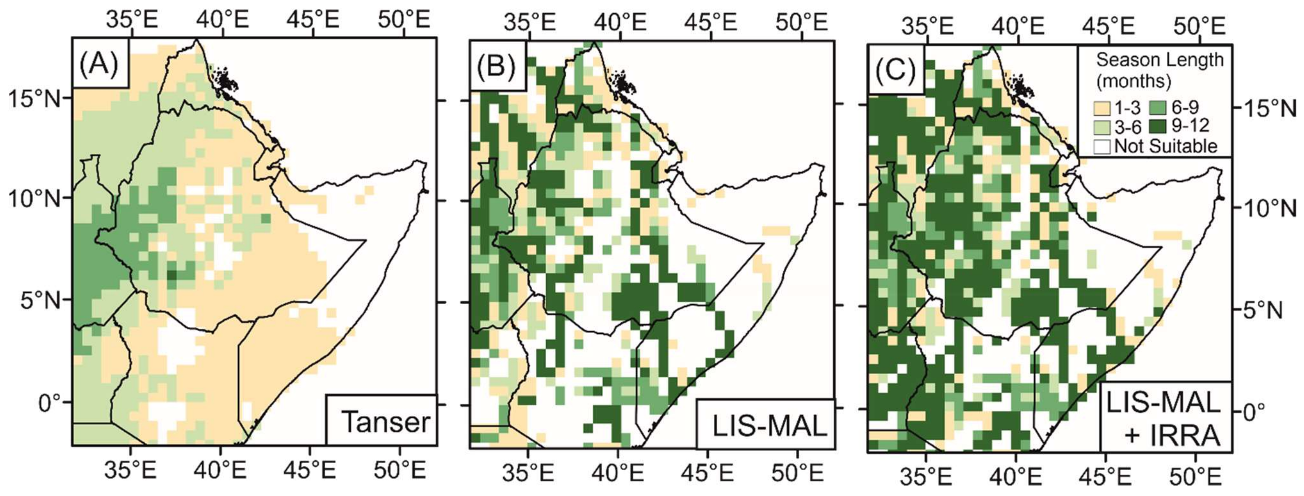

Supplementary Figure 20. **Comparison of hydro-climatic model estimates for Ethiopia and the Horn of Africa.**

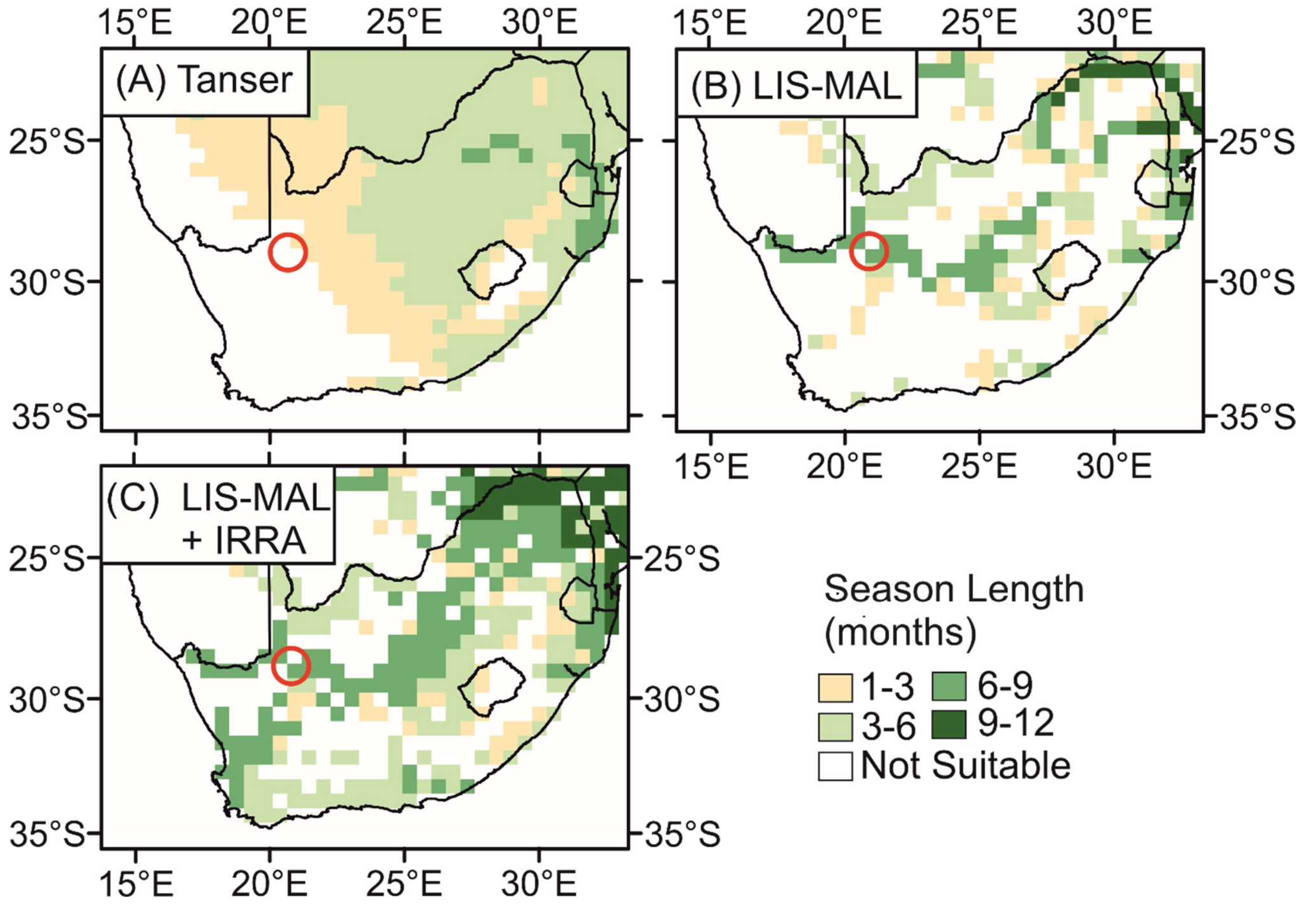

Supplementary Figure 21. **Comparison of hydro-climatic model estimates for South Africa.** The red circle indicates an area of discussion in Supplementary Note 2.

Supplementary References

[1] Gething, P. *et al.* Climate change and the global malaria recession. *Nature* **465**, 342–345 (2010).

[2] Lysenko, A. J. & Semashko, I. N. [in Russian] in Itogi Nauki: Medicinskaja Geografija (ed. Lebedew, A. W.) 25–146 (Academy of Sciences, Moscow, 1968).

[3] Reiter, P. Global warming and malaria: knowing the horse before hitching the cart. *Malaria Journal* **7**, S3 (2008).

[4] Kyalo, D. *et al.* A geo-coded inventory of anophelines in the Afrotropical Region south of the Sahara: 1898-2016. *Wellcome Open Research* **2**, 57 (2017).

[5] Bhatt, S. *et al.* The effect of malaria control on *Plasmodium falciparum* in Africa between 2000 and 2015. *Nature* **526**, 207-211 (2015).

[6] Wiebe, A. *et al.* Geographical distributions of African malaria vector sibling species and evidence for insecticide resistance. *Malaria Journal* **16**, 85 (2017).

[7] Kiszewski, A. *et al.* A global index representing the stability of malaria transmission. *American Journal of Tropical Medicine and Hygiene* **70**, 486−498 (2004).

[8] Ermert, V., Fink, A.H., Jones, A.E. & Morse, A.P. Development of a new version of the Liverpool Malaria Model. II. Calibration and validation for West Africa. *Malaria Journal* **10**, 62 (2011).

[9] Martens, W.J.M., Niessen, L.W., Rotmans, J. & McMichael, A.J. Potential impacts of global climate change on malaria risk. *Environmental Health Perspectives* **103**, 458−464 (1995).

[10] Tanser, F., Sharp, B.L. & Le Sueur, D. Potential effect of climate change on malaria transmission in Africa. *The Lancet* **362**, 1792–9178 (2003).

[11] Craig, M.H., Snow, R.W. & Le Sueur, D. Climate-based distribution model of malaria transmission in sub-Saharan Africa. *Parasitology Today* **15**, 105−111 (1999).

[12] Garnham, P.C.C. The incidence of malaria at high altitudes. *Journal of the Malaria Society* **7**, 275–284 (1948).

[13] Parham, P.E. & Michael, E. Modeling the effects of weather and climate change on malaria transmission. *Environmental Health Perspectives* **118**, 620–626 (2010).

[14] Lindsay, S.W., Parson, L. & Thomas, C.J. Mapping the ranges and relative abundance of the two principal African malaria vectors, *Anopheles gambiae* sensu stricto and *An. arabiensis*, using climate data. *Proceedings of the Royal Society B: Biological Sciences* **265**, 847–854 (1998).

[15] Daniels, R.F. *et al.* Evidence of non-Plasmodium falciparum malaria infection in Kédougou, Sénégal. *Malaria Journal* **16**, 9 (2017).

[16] Ndiath, M.O. *et al.* Low and seasonal malaria transmission in the middle Senegal River basin: identification and characteristics of Anopheles vectors. *Parasites & Vectors* **5**, 21. (2012).

[17] Dia, I. *et al.* Bionomics of malaria vectors and relationship with malaria transmission and epidemiology in three physiographic zones in the Senegal River Basin. *Acta Tropica* **105**, 145-153. (2008).

[18] Diouf, I. *et al.* Comparison of malaria simulations driven by meteorological observations and reanalysis products in Senegal. International *Journal of Environmental Research and Public Health* **14**, 1119 (2017).

[19] Trape, J.F. *et al.* Malaria morbidity and pyrethroid resistance after the introduction of insecticide-treated bednets and artemisinin-based combination therapies: a longitudinal study. *The Lancet Infectious Diseases* **11**, 925-932 (2011).

[20] Niang, E.H.A. *et al.* Malaria transmission pattern in an area selected for clinical trials in the Sudanian area of Senegal (West Africa). *Journal of Tropical Medicine* **2013**, 907375 (2013).

[21] Gemperli, A. *et al.* Spatial patterns of infant mortality in Mali: the effect of malaria endemicity. *American Journal of Epidemiology* **159**, 64-72 (2004).

[22] Coulibaly, D. *et al.* Spatio-temporal analysis of malaria within a transmission season in Bandiagara, Mali. *Malaria Journal* **12**, 82 (2013).

[23] Bruce-Chwatt, L.J. Malaria in Nigeria. *Bulletin of the World Health Organization* **4**, 301 (1951).

[24] Tolulope, O. Spatio–Temporal Clustering of Malaria Morbidity in Nigeria (2004–2008). *J Sci Res* **13**, 99-113 (2014).

[25] Labbo, R. *et al.* Longitudinal follow-up of malaria transmission dynamics in two villages in a Sahelian area of Niger during a nationwide insecticide-treated bednet distribution programme. *Medical and Veterinary Entomology* **26**, 386-395 (2012).

[26] Guillebaud, J. *et al*. Epidemiology of malaria in an area of seasonal transmission in Niger and implications for the design of a seasonal malaria chemoprevention strategy. *Malaria Journal* **12**, 379 (2013).

[27] Malik, E.M. & Khalafalla, O. Malaria in Sudan: Past, present and the future. *Gezira Journal of Health Sciences* **1**, 47-51 (2004).

[28] Ageep, T.B. *et al.* Spatial and temporal distribution of the malaria mosquito *Anopheles arabiensis* in northern Sudan: influence of environmental factors and implications for vector control. *Malaria Journal* **8**, 123 (2009).

[29] Hussien, H.H. Malaria's association with climatic variables and an epidemic early warning system using historical data from Gezira State, Sudan. *Heliyon* **5**, e01375 (2019).

[30] Nerlich, A.G., Schraut, B., Dittrich, S., Jelinek, T. & Zink, A.R. *Plasmodium falciparum* in ancient Egypt. [letter]. *Emerg Infect Dis.* **14**, 1317-1319 (2008).

[31] Kenawy M.A. Anopheline mosquitoes (Diptera: Culicidae) as malaria carriers in A.R. Egypt History and present status. *Journal of the Egyptian Public Health Association* **63**, 67– 85 (1988).

[32] Shousha, A.T. The Eradication of *Anopheles gambiae* from Upper Egypt, 1942-1945. *Bull. World Health Organ.* **1**, 309–352 (1948).

[33] Hassan, A.N., Kenawy, M.A., Kamal, H., Abdel Sattar, A.A. & Sowilem, M.M. GIS-based prediction of malaria risk in Egypt. *EMHJ-Eastern Mediterranean Health Journal* **9**, 548-558 (2003).

[34] Messina, J.P. *et al.* Population, behavioural and environmental drivers of malaria prevalence in the Democratic Republic of Congo. *Malaria Journal* **10**, 161 (2011).

[35] Janko, M.M. *et al.* The links between agriculture, Anopheles mosquitoes, and malaria risk in children younger than 5 years in the Democratic Republic of the Congo: a population-based, cross-sectional, spatial study. *The Lancet Planetary Health* **2**, e74-e82 (2018).

[36] PNLP, KSPH, Swiss KSPH, INRB and INFORM. *An epidemiological profile of malaria in the Democratic Republic of Congo*. A report prepared for the Federal Ministry of Health, Democratic Republic of Congo, the Roll Back Malaria Partnership and the Department for International Development, UK. September, 2014 (2014).

[37] National Malaria Control Team, Ethiopian Public Health Institute, World Health Organization, Addis Ababa University and the INFORM Project. *An epidemiological profile of malaria in Ethiopia*. A report prepared for the Federal Ministry of Health, Ethiopia, the Roll Back Malaria Partnership and the Department for International Development, UK. March, 2014 (2013).

[38] Noor, A.M. *et al.* Spatial prediction of *Plasmodium falciparum* prevalence in Somalia. *Malaria Journal* **7**, 159 (2008).

[39] Omer S.M. & Cloudsley-Thompson, J.L. Survival of female *Anopheles gambiae* Giles through a 9-month dry season in Sudan. *Bull World Health Organ*. **42** 319-30 (1970).

[40] Sharp, B.L. & Le Sueur, D. Malaria in South Africa the past, the present and selected implications for the future. *South African Medical Journal* **86**, 83-89 (1996).

[41] Kleinschmidt, I., Sharp, B., Mueller, I. & Vounatsou, P. Rise in malaria incidence rates in South Africa: a small-area spatial analysis of variation in time trends. *American Journal of Epidemiology* **155**, 257-264 (2002).

[42] Pratt-Johnson, M.J. The distribution of malaria in South Africa and a mosquito survey of military hospital areas. *Epidemiology & Infection* **19**, 344-349 (1921).

[43] Lindsay, S.W. & Martens, W.J.M. Malaria in the African Highlands: past, present and future. *Bulletin of the World Health Organization* **76**, 33−45 (1998).

[44] Martens P. *et al.* Climate change and future populations at risk of malaria. *Global Environmental Change* **9**, S89–S107 (1999).

[45] Martens, W.J.M., Jetten, T.H. & Focks, D.A. Sensitivity of malaria, schistosomiasis and dengue to global warming. *Climatic Change* **35**, 145–156 (1997).

[46] Hay, S.I. *et al.* Climate change and the resurgence malaria in the East African highlands. *Nature* **415**, 905−909 (2002).

[47] Alfieri, L. *et al.* Global projections of river flood risk in a warmer world. *Earth's Future* **5**, 171-182 (2017).

[48] Mordecai, E.A. *et al.* Optimal temperature for malaria transmission is dramatically lower than previously predicted. *Ecology Letters* **16**, 22-30 (2012).
