# Supplementary material for: Incorporating hydrology into climate suitability models changes projections of malaria transmission in Africa
Source: Nat Commun. 2020 Aug 28;11:4353. doi: 10.1038/s41467-020-18239-5 (PMC7455692; doi:10.1038/s41467-020-18239-5)
Supplement: Supplementary file 3 — Reporting Summary [file 41467_2020_18239_MOESM3_ESM.pdf]

natureresearch

Corresponding author(s): Mark Smith

Last updated by author(s): Jul 24, 2020

Reporting Summary

Nature Research wishes to improve the reproducibility of the work that we publish. This form provides structure for consistency and transparency in reporting. For further information on Nature Research policies, see Authors & Referees and the Editorial Policy Checklist .

Statistics

For all statistical analyses, confirm that the following items are present in the figure legend, table legend, main text, or Methods section.

n/a	Confirmed	
[x]	[ ]	The exact sample size (*n*) for each experimental group/condition, given as a discrete number and unit of measurement
[x]	[ ]	A statement on whether measurements were taken from distinct samples or whether the same sample was measured repeatedly
[x]	[ ]	The statistical test(s) used AND whether they are one- or two-sided<br>*Only common tests should be described solely by name; describe more complex techniques in the Methods section.*
[x]	[ ]	A description of all covariates tested
[x]	[ ]	A description of any assumptions or corrections, such as tests of normality and adjustment for multiple comparisons
[ ]	[x]	A full description of the statistical parameters including central tendency (e.g. means) or other basic estimates (e.g. regression coefficient) AND variation (e.g. standard deviation) or associated estimates of uncertainty (e.g. confidence intervals)
[x]	[ ]	For null hypothesis testing, the test statistic (e.g. *F*, *t*, *r*) with confidence intervals, effect sizes, degrees of freedom and *P* value noted<br>*Give P values as exact values whenever suitable.*
[x]	[ ]	For Bayesian analysis, information on the choice of priors and Markov chain Monte Carlo settings
[x]	[ ]	For hierarchical and complex designs, identification of the appropriate level for tests and full reporting of outcomes
[x]	[ ]	Estimates of effect sizes (e.g. Cohen's *d*, Pearson's *r*), indicating how they were calculated

Our web collection on statistics for biologists contains articles on many of the points above.

Software and code

Policy information about availability of computer code

Data collection	Hydrological analysis was performed using the freely available hydrological model Lisflood (https://ec.europa.eu/jrc/en/publication/eurscientific-and-technical-research-reports/lisflood-distributed-water-balance-and-flood-simulation-model-revised-user-manual-2013). The LISFLOOD model code is available for download here https://github.com/ec-jrc/lisflood-code.
Data analysis	Daily outputs from Lisflood were aggregated to the monthly scale over the date ranges used in this study. Python scripts for extracting monthly hydro-climatic variables from daily NetCDF climate data are provided here: https://doi.org/10.5518/786. Subsequent data analysis was undertaken in Stata 12.1

For manuscripts utilizing custom algorithms or software that are central to the research but not yet described in published literature, software must be made available to editors/reviewers. We strongly encourage code deposition in a community repository (e.g. GitHub). See the Nature Research guidelines for submitting code & software for further information.

Data

Policy information about availability of data

All manuscripts must include a data availability statement. This statement should provide the following information, where applicable:

- Accession codes, unique identifiers, or web links for publicly available datasets
- A list of figures that have associated raw data
- A description of any restrictions on data availability

Data used are as described by Alfieri et al. (2017) https://doi.org/10.1002/2016EF000485 and are available here: https://data.jrc.ec.europa.eu/collection/floods

The LISFLOOD model code is available for download at https://github.com/ec-jrc/lisflood-code, together with the pre-processor Lisvap used to calculate potential evapo-transpiration. Climate projections were provided by the Swedish Meteorological and Hydrological Institute in the context of the HELIX Project https://www.helixclimate.eu/wp-content/uploads/2018/04/HELIX-603864-D3.1-Documentation-of-changes-in-climate-variability-and-extremes-simulated-by-the-HELIX-

AGCMs-copy.pdf.
SPOT-VGT data was downloaded from http://wdc.dlr.de/data_products/
Hydrosheds elevation data from https://www.hydrosheds.org/downloads,
SoilGrids Database from https://www.isric.org/explore/soilgrids,
Channel geometry data through an email request to the corresponding author of Wu et al. (2012) as cited in the text.

LIS-MAL estimates of hydro-climatic suitability for malaria transmission in Africa (1971-2100) can be downloaded here https://doi.org/10.5518/786. The source data underlying all Figures are provided as a Source Data file.

Field-specific reporting

Please select the one below that is the best fit for your research. If you are not sure, read the appropriate sections before making your selection.

☐ Life sciences ☐ Behavioural & social sciences ☒ Ecological, evolutionary & environmental sciences

For a reference copy of the document with all sections, see nature.com/documents/nr-reporting-summary-flat.pdf

Ecological, evolutionary & environmental sciences study design

All studies must disclose on these points even when the disclosure is negative.

Study description	This study uses a hydrological model coupled with established temperature suitability ranges to provide a new map of hydro-climatic suitability for malaria transmission across Africa and estimate changes in that suitability up to 2100
Research sample	Lisflood hydrological simulations for 1971-2100 were provided by the corresponding author of Alfieri et al. (2017) as cited in the text. Date accessed: 6th February 2019
Sampling strategy	The land surface of Africa was sampled at a spatial resolution of 0.35 degrees
Data collection	Lisflood hydrological simulations for 1971-2100 were provided by the corresponding author of Alfieri et al. (2017) as cited in the text. Date accessed: 6th February 2019
Timing and spatial scale	Daily model timesteps are run between 1971 and 2100
Data exclusions	None
Reproducibility	The hydrological model was run using forcing data derived from seven independent driving GCMs produced within the Coupled Model Intercomparison Project Phase 5. Results from all seven are included herein.
Randomization	This is not an experimental study
Blinding	This is not an experimental study

Did the study involve field work? ☐ Yes ☒ No

Reporting for specific materials, systems and methods

We require information from authors about some types of materials, experimental systems and methods used in many studies. Here, indicate whether each material, system or method listed is relevant to your study. If you are not sure if a list item applies to your research, read the appropriate section before selecting a response.

Materials & experimental systems

n/a	Involved in the study	
☒	☐	Antibodies
☒	☐	Eukaryotic cell lines
☒	☐	Palaeontology
☒	☐	Animals and other organisms
☒	☐	Human research participants
☒	☐	Clinical data

Methods

n/a	Involved in the study	
☒	☐	ChIP-seq
☒	☐	Flow cytometry
☒	☐	MRI-based neuroimaging
